# Understanding Navon: A detailed structural and conceptual analysis of a basic local–global task

**DOI:** 10.3758/s13423-025-02741-2

**Published:** 2025-10-20

**Authors:** Felix Schweigkofler, Sjoerd Stuit, Johan Wagemans, Tanja Nijboer, Leendert van Maanen, Stefan van der Stigchel

**Affiliations:** 1 Utrecht University, Heidelberglaan 1, 83508 TC Utrecht, The Netherlands; 2 Katholiekee Universiteit Leuven, Tiensestraat 102 - Box 3711, 3000 Louvain, Belgium

**Keywords:** Visual perception, Navon task, Local–global bias

## Abstract

When perceiving visual information, either the local parts or more commonly the global whole can dominate on a perceptual-cognitive level (local/global bias). Using hierarchical figures consisting of smaller local elements forming a larger global shape, researchers have tried to understand how this local–global bias emerges. However, despite extensive research, local–global biases remain an elusive concept (possibly partially due to inadequacies of the task design itself), with implicit assumptions untested and different effect metrics being in use. To provide conceptual clarity, this study presents a detailed description of metrics and effects (content validity) and discusses four major assumptions in the current literature. Where possible, these assumptions are tested on a basic local–global task (the so-called Navon task) across 18 datasets (> 8,000 participants). The metric reliability is tested on nine datasets (> 7,000 participants). Our conceptual reasoning attempts to disentangle the complexity of interference effects in hierarchical figure tasks and discusses a potential facilitation effect. Our empirical results most importantly indicate: (1) the independence of the biased precedence and biased interference metrics, underlining that local–global biases do not reflect a unitary construct, (2) the independence of local-to-global and global-to-local interference, and (3) a low split-half reliability for interference metrics. Based on these insights we argue for further re-evaluating the concept (and theory) of local–global biases, prioritizing research on a thorough conceptual (and theoretical) understanding of local–global tasks, a more consistent use of bias-related metrics in future studies, and a possible need for a more mechanistic approach to facilitate effective future research on hierarchical figure tasks.

**Supplementary Information:**

The online version contains supplementary material available at 10.3758/s13423-025-02741-2.

## Introduction

Visual information is processed from a set of discrete inputs on the retinae that are progressively and hierarchically integrated into a meaningful whole (Palmer, 1999). On a higher, perceptual level, this integration process takes the form of integrating local spatial elements into a global shape, for example, integrating the representations of individual local keys of a keyboard into a more global representation of the keyboard as a whole. Whether the information from the local or the global level is perceived more quickly and/or accurately during a cognitive task depends on the design of the figure and the task procedure as well as the state and trait bias of the participant (de-Wit & Wagemans, 2014). Note that the term *bias* in the context of past and current local–global research purely expresses the difference in observed responses. The term has no mechanistic or other implications and does not refer to a deliberate strategy to conduct the task. Rather, the bias should be understood as the effects on the level of attention or perception.

There is currently still no clear consensus in the literature regarding what cognitive mechanism underlies a possible bias towards local or global processing (de-Wit & Wagemans, 2014; Navon, 2003). However, it has become clear that individual differences are significant, as bias metrics are altered in different clinical conditions, such as autism (Baisa et al., 2021; Hayward et al., 2018; Van der Hallen et al., 2015), anxiety (Kalanthroff, 2023; Retzler & Retzler, 2023), schizophrenia (Kurylo et al., 2007), and anorexia nervosa (Weinbach et al., 2017).

**Fig. 1:**
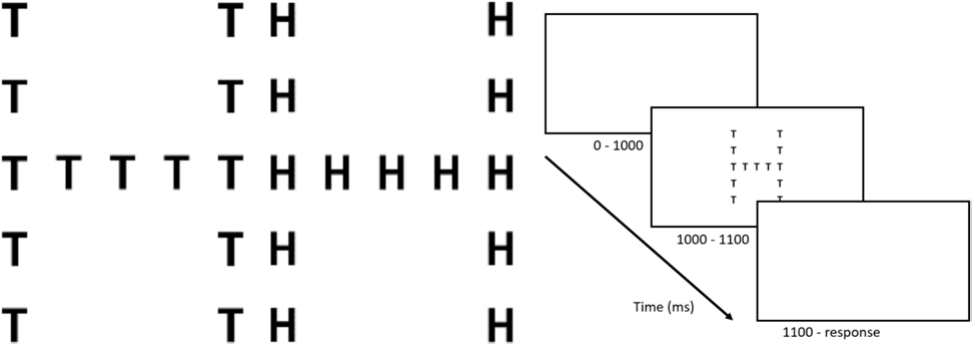
A simple selective-attention local–global task: The hierarchical compound figure is incongruent (left) when the identity of the local element (here: T) differs from the identity of the global figure (here: H) and congruent (centre) when they are the same (here: both H). The participant is instructed beforehand on which level (local or global) to focus and to report the target’s identity as quickly as possible by pressing the corresponding key on the keyboard. Usually, an instruction is followed by a block of dozens of trials. The figures can be altered in many aspects, such as size and number of the local elements, density of the local elements, familiarity of the item (letter or shape) to be reported, number of possible items to be differentiated, etc. This specific task design is commonly referred to as *Navon task*

The local–global bias is often examined using figures that depict a global shape consisting of (congruent or incongruent) local elements (compare Fig. 1). Ever since their introduction by Asch in the mid-twentieth century (Asch, 1962), these so-called *hierarchical compound figures* have been used for testing a wide variety of hypotheses, for example in a recall-setting (Asch & Ebenholtz, 1962), for visual scanning (Neisser, 1976), or for local target detection (Kinchla, 1974, 1977). Even in more recent literature researching explicitly the local–global bias within the current theoretical framework, varying designs are in use (Behrmann et al., 2005; Morris et al., 2021; Poirel et al., 2008). In local–global research, the specific task procedure employing these hierarchical figures that is most frequently used is usually referred to as the *Navon task* (Navon, 1977). For terminological consistency, we use the term *Navon task* throughout this article to refer to this particular task design, while we use the term *local–global* to refer to the paradigm more generally.

The Navon task is a simple local–global task in which participants are shown a hierarchical figure (Fig. 1) and according to prior instruction, a participant needs to report either the local or the global letter. Thus, *selective attention* should occur.

In the less frequently used divided-attention designs of local–global tasks, a trial’s target level is not known to participants beforehand so that both levels need to be attended. The *structural-conceptual* analysis of divided-attention designs is largely similar to that of selective-attention designs, but some conceptual and especially *theoretical* aspects may differ (e.g., the ability to focus solely on one level vs. the ability to quickly scan two levels). As this article deals with structural-conceptual analysis, we decided to focus on the simpler and more common selective-attention design. Due to the possibly significant theoretical difference between these two task designs, the *empirical* results (which are based on the Navon task) should only be generalised to divided-attention designs and to other (non-Navon) selective-attention designs with great caution, if at all. Even within the Navon design, small differences like letter density and exposure duration might have systemic effects; however, we believe the studies used in this article are broadly comparable for our intents and purposes.

Even when neglecting differences between designs, conceptual complexity remains, as local–global tasks using hierarchical figures contain a factor *Level* and a factor *Congruence*. Due to these two factors, multiple effect metrics can be calculated. These metrics are currently used inconsistently in the literature (see *Metrics in the literature*), leading to non-comparable empirical results and impeding progress in the field. This lack of conceptual and theoretical convergence was noted before (Navon, 2003) and is especially problematic because the specificity of many local–global hypotheses require more solid conceptual, empirical, and theoretical foundations than the field can currently offer. The use of the local–global paradigm in clinical settings (Akerman et al., 2023; Álvarez-San Millán et al., 2022b; Kalanthroff, 2023) underlines the urgent need for conceptual clarity.

In the current study, we therefore first provide a structural and conceptual description of the effect-metrics and discuss related conceptual assumptions, then present empirical findings about the reliability of metrics and the conceptual assumptions, and finally interpret the significance of these conceptual and empirical findings for past and future research.

At this point we also want to note that we explicitly differentiate between four different layers of abstraction throughout the paper (details are given in Online Supplemental Material, Appendix #1):

- ‘Structural’ refers to the purely mathematical relations.
- ‘Conceptual’ refers to all lower-order assumptions and interpretations related to and arising from basic aspects of the task itself.
- ‘Empirical’ refers to all observable results and real-world data.
- ‘Theoretical’ refers all higher-order interpretations that integrate conceptual reasoning and empirical results with cognitive theories (e.g., effect mechanisms).

## Effect metrics

### Bias effects

Conceptually speaking, the local–global bias has two different facets (Gerlach & Krumborg, 2014).

1. The *biased precedence effect* describes the general bias towards higher performance processing local or global information in the absence of any cross-level interference effects. In the Navon task, the target level is known.
2. The *biased interference effect* describes whether the interference of information from the incongruent non-target level (in incongruent trials, Fig. 1, left) is stronger when the target level is local or global.

A third effect – the *congruence effect* – is also sometimes described (Gerlach & Krumborg, 2014), but since it is not a level-bias effect, it is of less importance in the local–global literature.

The biased precedence and biased interference both describe *a* kind of local–global bias, but not necessarily *the same* kind of local–global bias. Navon himself already pointed out the existence of precedence and interference effects (Navon, 1977), but despite some standardization efforts (Gerlach & Krumborg, 2014), there is no consensus in the literature of how exactly these effects are extracted from the basic performance measures (reaction time or accuracy) gathered in the task (see *Metrics in the literature*). We will therefore outline the two task factors and how they form the structure of the metrics to provide a basis for our further conceptual analysis.

### Metrics

**Fig. 2:**
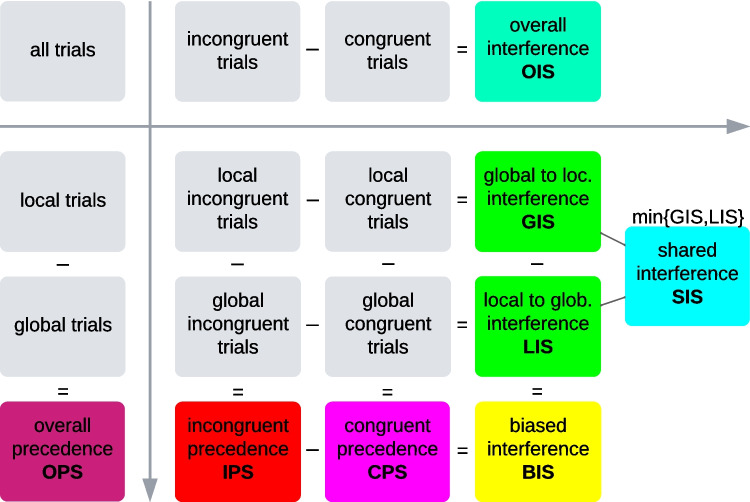
Calculation of eight Navon metrics (coloured blocks) from the trial conditions (grey blocks). Subtracting a summary performance measure (e.g., mean reaction time) of one trial condition (e.g., global trials) from another trial condition results in a metric-score. Note the two-dimensional layout: the horizontal dimension represents the task factor Congruence, and the vertical dimension represents the task factor Level/Precedence. Within this factorial design, OIS and OPS are main effect metrics, GIS, LIS, CPS, IPS are simple main effect metrics, BIS is the interaction effect metric, and SIS is an additional effect metric outside of this traditional statistical effect framework. Crucially, local–global bias (effect of Level/Precedence) strictly refers to the difference along the y-axis, while the difference along the x-axis denotes Interference (effect of Congruence). As is common in the literature, we subtract global from local (left to right in Fig. 2) and congruent from incongruent. If the direction of calculation were to be reversed, the absolute value of the metrics would still be the same, but their sign would be reversed. For an example calculation with hypothetical values, see Online Supplemental Material, Appendix #2 (Fig. 10). Note that the calculation framework is inherently agnostic to the type of measure, but the conceptual and theoretical interpretation of resulting metrics is not (see Online Supplemental Material, Appendix #5)

Trials in the Navon task can be classified by the factor *Target Level* (global or local level) and the factor *Congruence* (congruent or incongruent figure), leading to four different trial types in a 2 × 2 factorial design. These four trial types can be used to calculate a wide range of possible effect metrics (Fig. 2), which can be split into four categories, namely *main* effect metrics, *simple main* effect metrics, the *interaction* effect metric (in reference to ANOVA effects), and *other* effect metrics (outside of the structure of ANOVA effects).

It is important to note that due to this effect structure, performing an ANOVA on the trial data is statistically closely related to calculating metrics from the trial data and performing a t-test on these metrics. Our conceptual analysis therefore applies to all local–global studies, regardless of whether they calculated metrics or used an ANOVA on trial types. Furthermore, we want to mention that the following explanation of effect metrics reflects the general use in the literature and requires that two assumptions (A1 and A2, see *Assumptions* section) hold true.

### Main effect metrics

When the congruence factor is ignored and all local trials are compared to all global trials, the resulting difference is the *overall precedence score* (OPS), which expresses whether a participant is faster on local or global trials. When the precedence factor is ignored and all incongruent trials are compared with all congruent trials, the resulting *overall interference score* (OIS) expresses whether a participant is faster on congruent or incongruent trials. However, simply ignoring one of the two task-factors in the calculation does not remove its statistical influence. OPS and OIS therefore do not capture a conceptually pure precedence and pure interference effect, but rather a mix of effects.

From a conceptual point of view, they are therefore generally inappropriate in terms of representing any of the core effects (biased interference, biased precedence, congruence effect), and we will not discuss them conceptually. However, as the OPS can be empirically related to other precedence metrics and is used in many studies, we will report its empirical properties and correlations. Note that in the special case that BIS = 0, CPS, IPS and OPS are identical (same for LIS, GIS, and OIS); in other words when the task factor Congruence is irrelevant, OPS is a valid representation of the factor Level (and vice versa for OIS).

### Simple main effect metrics

Splitting the trials with regard to both task factors (target level and congruence of target identity) results in four trial types (Fig. 2, centre). From these four trial types, the following four metrics can be calculated. Two theoretical assumptions that are necessary for this calculation are discussed in detail later.

The *congruent precedence score* (CPS) expresses the response bias towards either global or local trials for congruent stimuli. This metric assumes that in congruent trials no cross-level interference from the non-target level exists. We define cross-level interference here as the influence from the non-relevant level (e.g., the local level) on the target level (e.g., global level). If this assumption holds, the CPS captures a single, conceptually clear effect, the *biased precedence effect*; the sign of the score describes the direction of the bias. In our formulation this entails that negative means a local bias and positive means a global bias.

The *incongruent precedence score* (IPS) also expresses a local–global response bias, but for incongruent figures. Typically, it is assumed that cross-level interference from the incongruent information of the non-target level occurs. If such an interference did not occur, the IPS and CPS would be the same. When an experiment uses exclusively incongruent trials, as is frequently done (e.g., D’Souza et al., 2016; Lee et al., 2023; Noguchi & Tomoike, 2016), the IPS is the only metric that can be calculated.

The *global-to-local interference score* (GIS) expresses the interference-effect of global distractors (incongruent global information) on local processing as the difference between the performance in local congruent and local incongruent trials.

The *local-to-global interference score* (LIS) does the same, but for local distractors in global trials. Note that since GIS and LIS apply to only local and only global trials, respectively, these metrics do not capture *bias* effects and must in themselves be seen as pure Congruence effects.

We want to stress here that *bias* is a property of the precedence-dimension (see Fig. 2) and refers to the *observational* difference between the local or global condition. A pure Congruence effect in local–global tasks (e.g., global-to-local interference) is *mechanistically* the result of an interaction between local information and global information *within* a single trial. Therefore, pure Congruence alone is not itself a *bias* and only when pure Congruence in the local and global condition are compared, do we obtain a bias. This becomes evident when imagining a participant with a high GIS and high LIS: If GIS and LIS were bias effects, this participant would show a global and local *bias* simultaneously, which is against the definition of bias (see visualization in Online Supplemental Material, Appendix #3).

Importantly, it is widely assumed that incongruent trials are always slower or maximally equally as fast as congruent trials and therefore GIS and LIS should always be positive values. However, negative scores do exist empirically (Fig. 6) (e.g., Chamberlain et al., 2017; Gerlach & Poirel, 2020), for reasons that are not fully clear. We discuss the conceptual implications of negative LIS and GIS in the *Assumptions* section (sub-section A1) and potential approaches to examine the empirical relevance of these negative scores in the *Results* section (sub-section A1).

To summarise, splitting the data by the factor Congruence and using the factor Level for the calculation results in CPS and IPS, which are bias metrics with no inherent information about interference. Doing the opposite results in GIS and LIS, which are interference metrics with no inherent information about the level bias.

### Interaction effect metrics

It is possible to combine both factors and obtain the interaction effect between Level and Congruence, the *biased interference score* (BIS), either by subtracting the CPS from the IPS or – perhaps more intuitively – by subtracting the LIS from the GIS. The resulting difference (BIS) expresses whether a participant experiences a stronger global-to-local interference or a stronger local-to-global interference. Positive BIS indicating a global bias is common in the literature (Avidan et al., 2011; Behrmann et al., 2005; Wang et al., 2012; Wong et al., 2021).

It is important to note that BIS captures a conceptually relevant bias effect, but its theoretical worth is not clear, as it could merely be the sum of a number of underlying mechanistic (theoretical) effects. In particular, global-to-local interference and local-to-global interference could be based on entirely separate cognitive mechanisms that merely sum up to an observational bias without inherent theoretical significance. For the BIS to have theoretical value, it seems important that a singular cognitive interference-mechanism exists. In that case BIS is not merely an observational effect, but a full description of the interference effect, and GIS and LIS would be two sides of the same coin. However, as the theoretical understanding of these effects is currently so limited and any relationship between global-to-local and local-to-global interference is imaginable, these statements should be seen as mere conjectures.

At this point it is also important to briefly revisit the case of negative GIS and/or LIS: Calculating BIS by subtracting LIS from GIS means that *negative* global-to-local interference scores are effectively treated as if they were *positive* local-to-global interference scores (i.e., − GIS ≙ + LIS) and vice versa. This has specific conceptual implications that we discuss in the section *Incongruent interference assumption*.

### Other effect metrics

As only positive GIS and LIS are conceptually expected (A1: *Incongruent interference assumption*, see below), the smaller of the two interference scores can be viewed as a kind of level-neutral, non-biased, ‘baseline’ interference that is shared between the two metrics. Thus, we introduce the *shared interference score* (SIS) as a (to our knowledge) new metric that is defined as the smaller one of GIS and LIS. Conceptually, SIS is in principle the proper way of expressing the non-biased interference effect, also referred to as Congruence effect (Gerlach & Krumborg, 2014). Traditionally, the Congruence effect is expressed with the mixed-effect metric OIS that merely averages over global and local trials and therefore contains the biased interference next to the shared interference (for empirical relations, see Fig. 4). Empirically, the correlation between OIS and SIS may be high (Fig. 4), but we wanted to discuss the principal conceptual difference nevertheless.

It is important to note that while SIS (and OIS) has a clear *conceptual* interpretation as a non-biased interference effect, it is not clear what its *theoretical* value is. A “higher proneness to any cross-level interference” may or may not be a relevant mechanistic feature of a participant’s perceptive system. The interpretation of SIS in particular is influenced by whether global-to-local and local-to-global interference are rooted in one singular or two separate mechanisms.

For structural completeness and to underline the difference between structural and conceptual analysis, we want to mention the “shared precedence score (SPS)” as well: One could apply the structural methodology of SIS to the precedence metrics IPS and CPS to obtain the structurally symmetric ‘SPS’. However, since precedence is bi-directional and both positive and negative scores are expected and valid, this metric would just capture a participant’s “strongest locally biased score independent of congruence”, which by itself is probably not relevant information for most research questions. However, we would like to use the example of SIS and SPS to encourage researchers to not blindly stick to traditional metrics but rather to think in conceptual terms and come up with new metrics, if such alternative metrics seem to be more appropriate for the hypothesis at hand.

### Metrics summary

Conceptually speaking, CPS captures the biased precedence, SIS the level-neutral interference, and BIS the biased interference. For every participant, either GIS or LIS is equal to SIS (the interference ‘baseline’) and the other one captures a mix of SIS and BIS (importantly, this *conceptual* interpretation does not necessarily apply *theoretically*). IPS always captures a mix of CPS and BIS. OPS captures the average of CPS and IPS. OIS captures the average of GIS and LIS.

### Alternative calculation

So far, we have described the metric calculation as a simple subtraction of mean reaction times, but a division or even more complex operations are also imaginable. Additionally, the resulting metric could be normalised or standardised in some way, for example by dividing it through the participants overall reaction time. We discuss some of these options in more detail in Online Supplemental Material, Appendix #4 and want to focus here on the common *standardised mean difference* (SMD), also known as Cohen’s d.

When using the SMD, one calculates a simple difference of the means (as we do) but then divides it by the pooled standard deviation (SD) of the minuend and subtrahend. As SMD expresses an effect size as multiples of the SD, wider distributions lead to smaller values, i.e., when two participants have identical means in local and global reaction times, but different spreads, the SMD states that the participant with the wider spread has a smaller bias. The same logic applies metrics within a participant. Depending on the research question, this behaviour of SMD and its implications for the results-interpretation may or may not be desired.

For the local–global paradigm, we are not convinced that the SMD is necessarily more useful than the simple mean difference. However, examining the spread separately from the mean (not in a mixed summary like the SMD) might add explanatory value, since mean and spread have different theoretical implications. In conclusion, we recommend that the SMD should only be used after a careful consideration and ideally next to the simple difference, not instead of it.

A further possible divergence in analysis is in the kind of measure that is used. The measure of choice is generally the reaction time, but the error rate or any other measure collected during the experiment could be used as well, as the calculation of effects is input-agnostic. However, to interpret such metrics the conceptual and theoretical implications of the underlying measure need to be considered (example error rate in Online Supplemental Material, Appendix #5). To keep our analysis focused we use the reaction time, which is the most straight-forward measure. However, relevant insights may be gained using error rates and we do not intend to discourage the use of alternative measures.

### Metrics in the literature

The CPS, SIS and BIS correspond to the three conceptual effects that have been identified in the literature (Gerlach & Krumborg, 2014): biased precedence, shared interference and biased interference. However, not all studies differentiate between the biased precedence and biased interference effect, and when they do, these effects are often named differently and/or linked to the wrong metrics. We want to show a few examples here:

Some studies use only reaction-time-based LIS and GIS (English et al., 2017; Kalanthroff, 2023; Weinbach et al., 2017) or GIS alone (Dale & Arnell, 2013), while others use reaction-time-based and accuracy-based GIS and LIS in addition to reaction-time-based CPS (Hedge et al., 2018), but CPS has also been used alone (Gerlach et al., 2017), or together with IPS (Álvarez-San Millán et al., 2022a), or together with IPS and GIS (Duchaine et al., 2007a, 2007b). Reaction-time-based and accuracy-based IPS have also been used together with OPS (Hayward et al., 2018), whereby the former was used to express ‘global interference’ and the latter ‘global advantage’, which is not a conceptually accurate description of these metrics according to our reasoning. The interference bias should be better represented by BIS, which was used by several studies as well (Avidan et al., 2011; Behrmann et al., 2005) and has been inaccurately named “global-to-local interference” by others (Wang et al., 2012; Wong et al., 2021). One study calculated the OPS as a ratio between global and local trials (Duchaine et al., 2007a, 2007b) and not as a difference.

## Assumptions

The inconsistent use of effects and metrics throughout the literature is indicative of a broader underlying issue in the conceptual understanding and interpretation of local–global tasks in the literature. This issue also extends to the existence of several critical but usually implicit conceptual assumptions regarding the calculation or interpretation of Navon metrics. We think that four of these implicit assumptions are particularly important for a thorough understanding of the Navon task. Please note that Assumptions 1 and 2 are a priori theoretical assumptions that underly the very calculation of bias scores as outlined in Fig. 2, while Assumptions 3 and 4 are a posteriori assumptions about the metrics that are mostly relevant for their theoretical interpretation and can more easily be tested empirically. In the following sections we elaborate on these assumptions from a conceptual perspective.

### Incongruent interference assumption

Cross-level *interference* occurs on incongruent trials but not on congruent trials, cross-level *facilitation* does not occur in local–global paradigms.

Cross-level influence refers to the influence of one level of a compound figure on the processing of the other level, be that a negative (interference) or a positive influence (facilitation). Particularly relevant in selective-attention local–global tasks like the Navon task is the influence of the predefined non-target level on the predefined target level.

The central assumption about congruence in local–global tasks is that only interference in incongruent trials is relevant and all other cross-level influences can be neglected. That means that congruent trials are assumed to act as true neutral trials (free of congruence effects) to which incongruent trials can be compared to extract the incongruent interference effect. Congruent facilitation is rarely explicitly discussed in local–global literature and is largely assumed to be inexistent or irrelevant.

The *incongruent interference assumption* is crucial for the conceptual (content) validity of the interference metrics, as the congruent value is subtracted from the incongruent value precisely to get at this non-target-to-target interference in incongruent trials – the interference effect (OIS in all trials, GIS in local trials, or LIS in global trials, BIS as difference between GIS and LIS). If other cross-level influences did not exist, this would conceptually complicate the calculation and interpretation of these metrics as well as the interpretation of IPS and CPS (of course the actual relevance depends on the empirical magnitude of these other influence effects). We will therefore first conceptually explain all four potential effects and their implications and later try to establish their (non)-existence empirically.

**Fig. 3:**
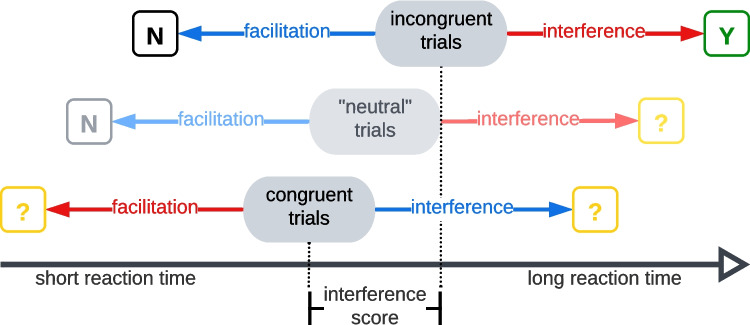
Hypothetical cross-level influence effects. Incongruent trials tend to be slower than congruent trials or ‘neutral’ trials. As GIS and LIS are calculated as *incongruent – congruent* (for local and global trials, respectively), cross-level influence effects can cause GIS and LIS to be positive (effects marked in red) or negative (effects marked in blue). We suspect that the ‘neutral’ trials that are used in the literature are actually just a variety of incongruent trials (visualised as a shift towards greater interference). Hypothetical *true* neutral trials are characterised by an absence of any influence effects (not depicted). In the current literature, congruent trials are commonly implicitly treated as true neutral trials, hence their use as subtrahend in the calculation of interference scores. The markers Y, N,? indicate whether an effect is conceptually sensible, not sensible, or uncertain

In both global and local trials incongruent and congruent figures exist (Fig. 3) and in both Congruence conditions the non-target information can hypothetically influence the processing of the target-level information and thereby influence the participant’s measurable response.

*Interference in incongruent trials* (top, red in Fig. 3) means that information from the non-target level (e.g., H) interferes with the processing of incongruent information from the target-level (e.g., T). This is the standard effect assumed throughout the literature.

*Facilitation in incongruent trials* (top, blue in Fig. 3) means that the information from the non-target level (e.g., H) decreases the reaction time of the incongruent target-level information (e.g., T). However, while *gradual* and *dependent* information can be enhanced by incongruent information (e.g., a blue target appearing even bluer when surrounded by a yellow non-target), such an enhancement seems implausible for *categorical* and *independent* information like the letters and shapes in a local–global task.

*Facilitation in congruent trials* (bottom, red in Fig. 3) means that the information from the non-target level (e.g., H) improves the processing of the congruent target level information (e.g., H). Since it is the same information, this effect is plausible, especially when the participant has a bias towards the current non-target level. This effect could be comparable to the facilitation effect in the Stroop task (MacLeod, 1991; van Maanen et al., 2009).

*Interference in congruent trials* (bottom, blue in Fig. 3) means that congruent information from the non-target level interferes with the processing of the target level information. Such congruent interference would likely be due to the existence of *any* interpretable information on the non-target level and not so much a specific relationship with the information on the target level. Such an effect was discussed previously in the context of the Stroop task (Entel et al., 2015) and referred to as ‘negative facilitation’, i.e. interference.

In summary, incongruent interference is conceptually sensible, incongruent facilitation is not, and both interference and facilitation might exist in congruent trials, which would undermine the *incongruent interference assumption*. Metrics that are directly or indirectly based on congruent trials (all except IPS) would thus contain this congruent influence effect in some form and the single-effect metrics CPS, BIS and SIS would all be mixed-effect metrics. The existence of cross-level influence in congruent trials would thus have major implications for the conceptual interpretation of local–global tasks.

A hint for the empirical existence of congruent *interference* in particular is that for some participants, the mean congruent trial is slower than the mean incongruent trial, leading to negative GIS/LIS (Fig. 6) (e.g., Chamberlain et al., 2017; Gerlach & Poirel, 2020). The only conceptual explanation for this behaviour is *congruent interference* (incongruent facilitation is implausible), but noise can be an alternative, empirical explanation. We discuss the empirical perspective in the *Results* section and focus here on an alternative approach: neutral trials.

Hypothetical true neutral trials are defined by the absence of all cross-level influence effects and are therefore the most direct way to test whether congruent *net influence* effects exist. For other interference paradigms such as the Stroop task (Klein, 1964; MacLeod, 1991) or the picture-word interference paradigm (Glaser & Düngelhoff, 1984; van Maanen et al., 2012), it was empirically shown that trials with a (more) neutral design have less interference.

For the Navon task, researchers tried to make non-target information less salient and distracting by using response-irrelevant information, such as an X or # to which the participant never had to and could not even respond to (Álvarez-San Millán et al., 2021, 2022a, 2022b; Martin, 1979; Morris et al., 2021; Van Kleeck, 1989; Wong et al., 2021). One researcher removed the global information altogether by presenting a single local element (Álvarez-San Millán et al., 2021, 2022b, 2022a), raising the question of whether this is still a local–global stimulus. Aside from that, we are not convinced that these designs represent or come close to a true neutral condition, since # and X are still written symbols that do not seem to be much less salient than other letters in a task as fast as the Navon task (Fig. 3, middle). While these ‘neutral’ trials and congruent trials were found to be similar in reaction time and accuracy in a study using shape stimuli (Morris et al., 2021), several studies using letter stimuli report (much) longer reaction times for the ‘neutral’ than the congruent trials (Álvarez-San Millán et al., 2021, 2022b; Van Kleeck, 1989).

This suggests either that these ‘neutral’ trials are similar to incongruent trials, or that congruent facilitation exists, or both. In either case, we argue that our understanding of congruent facilitation (and interference) in the local–global paradigm is insufficient. For our further analyses, we assume that the *incongruent interference assumption* and therefore our metric-effect-description are broadly accurate. If future research disproves this assumption, results based in interference scores need to be reinterpreted.

### Precedence-interference independence assumption

There is no synergy (i.e., interaction^1^) between congruent precedence and biased interference effects.

Assuming the *incongruent interference assumption* is valid, congruent trials capture the biased precedence effect and incongruent trials capture the biased precedence as well as the global-to-local or local-to-global interference effect. This means that interference effects never exist independent of the biased precedence and need to be calculated from the total observed effect and the biased precedence effect. This calculation requires an assumption on the synergy between biased precedence and interference effect. The most basic assumption is that the effects are ‘rigid’, i.e., do not interact. In that case, the biased precedence plus the interaction effect equals the total effect (CPS + BIS = IPS). This is the usual assumption in the literature and also the assumption we used in this article (see Fig. 2 specifically).

However, it could be that there exists a synergy of some kind between the two effects. An example for such a synergy would be a ceiling effect where a large ‘true’ biased precedence and a large ‘true’ interference effect do not add up linearly and the observable total effect is less than the sum of the two (i.e., a kind of ceiling effect). Assuming rigid effects would then lead to a distorted calculation of the interference effect.

From a conceptual perspective, the assumption about the precedence-interference synergy is necessarily an a priori assumption that cannot be determined within the conceptual analysis. It is thus up to theory or empirical research to answer this question. However, there is no clear theoretical reasoning for the kind of synergy one would expect and the widely assumed rigidity of effects (i.e., no synergy) seems like a reasonable assumption. It also seems very difficult to derive the nature of the synergy from empirical data due to circular reasoning (to calculate effects, an assumption must be made first) and because this synergy may be hard to distinguish from actual theoretical-mechanistic effects that we are interested in. In the *Results* section, we attempt to make a limited empirical inference, but for this article at large we assume that the effects are indeed rigid and subtraction of biased precedence from the total observable bias gives an appropriate interference effect size.

### Interference independence assumption

Global-to-local and local-to-global interference are independent metrics.

Other authors argued for treating local processing and global processing as *independent* cognitive mechanisms (Förster & Dannenberg, 2010), and GIS and LIS have been used as independent measures (Chamberlain et al., 2017; Gerlach & Poirel, 2020). Some authors have implicitly treated LIS and GIS as interchangeable metrics by using only one of them (Dale & Arnell, 2013). However, as knowledge about local–global processing mechanisms is very limited, treating the global-to-local interference (GIS) and local-to-global interference (LIS) as independent effects has currently no clear theoretical basis. It is important to remember that LIS and GIS are not *bias* metrics by themselves (see metrics description) and therefore any discussion about local–global *bias* necessarily needs to compare the two. The alternative line of thinking does not treat GIS and LIS as relevant effects, but rather their difference (BIS) and their shared baseline (SIS). This alternative view is best understood theoretically as a single mechanism that processes the local and global level and suffers from interference between the two but is more prone to interference in one direction than the other.

### Unitary bias assumption

Navon captures a singular local–global bias.

Another frequent assumption in local–global research is that there exists a (largely) unitary bias construct that can be captured by the Navon task (Chamberlain et al., 2017). However, if Assumptions 1 through 3 hold, there are two conceptually valid bias effects, the biased precedence effect and the biased interference effect. Depending on which singular metric is chosen by researchers to represent the assumed unitary bias, different formulations of the u*nitary bias assumption* are implied:

- When either CPS or BIS (or OPS) is used
  o it is either implied that biased precedence and biased interference are assumed to be highly correlated and therefore interchangeable (empirical implication),
  o or it is implied that the two are assumed to *not* be correlated but that only one of them is relevant (theoretical implication).
- When IPS is used, it is implied that biased precedence and biased interference by themselves do not represent ‘the bias’ and only a metric combining the two (IPS) should be used. This is in principle a theoretical implication, but since IPS is correlated with both BIS and CPS, it could also be based on pragmatic considerations by a researcher.
- When only GIS or LIS are used by themselves, there are strictly speaking no unitary bias implications, as GIS and LIS are not bias metrics (see section *Simple main effect metrics*). When both GIS and LIS are used and compared in the interpretation, this is essentially BIS and would imply points (a) and (b). Please note that analysis of GIS and LIS can be valid, just not independently as representation of a bias.

We will test the implied empirical assumption of point (a) by correlating BIS and CPS, but the implied assumptions of points (b) and (c) are theoretical conjectures and cannot easily be tested empirically with our data. As these assumptions are usually not explicit, we do not know of any specific hypotheses or statements that we could discuss from a conceptual perspective.

## Methods

For both our conceptual structural and empirical analyses we use the Navon task (Fig. 1) because it is the conceptually clearest and simplest version of local–global tasks that we know of (selective attention, single figure). It is also the most widely used version in the current literature, and we therefore expected the most success in gathering existing data.

### Search methods

For our empirical analysis, we gathered the raw data (reaction time and correctness per trial) or pre-processed data (reaction time and (not always) accuracy summarised by trial type) by performing a non-exhaustive, non-systematic Google Scholar search with the search query ‘*local* + *global* + *bias* + *navon’* over the course of autumn 2023. We mostly limited our search to papers published in and after 2015 to increase the chance of responses from researchers and the chances of finding Open Data. We excluded all non-Navon tasks and all Navon tasks without congruent trials (those studies can only use the IPS). We contacted the authors of 38 studies that had not made their data openly available.

### Obtained datasets

In addition to our own unpublished data (replication of Dale & Arnell, 2013) and six openly available datasets, we received 11 more datasets (including two with (partially) unpublished data), totalling 18 studies (Table 1) with data from Navon trials that follow a 2× 2 congruency × level design (congruent/incongruent × global/local). Some experiments used ‘congruence-neutral’ trials which we did not include in our analysis. For a discussion on these ‘neutral’ trials, see the *Incongruent interference assumption* and Fig. 3.

**Table 1:** Studies from which we obtained data named after their four-letter code from the *LoGlo-Repo* on OSF (https://osf.io/xtrhe/), their participant groups and task conditions, their tag in this paper, their participant number (N) and trial number (tr), as well as the overall accuracy (%) and mean reaction time (RT) in seconds

Study	Tag	Group	Condition	N	Tr	%	RT	Reference
EZIG	A1	Control	Auditory	18	160	69	3.98	(Akerman et al., 2023)
	A2	ADHD		14	160	67	4.43	
WHIK	B1	AN	None	17	192		0.62	(Weinbach et al., 2017)
	B2		Tone	17	192		0.58	
	B3	Control	None	21	192		0.61	
	B4		Tone	22	192		0.58	
SJIB	C1	ASD	Figures: Large	23	288	94	0.52	(Baisa et al., 2021)
	C2		Medium	25	288	94	0.50	
	C3		Small	23	288	93	0.52	
	C4	Control	Large	25	288	95	0.49	
	C5		Medium	25	288	95	0.48	
	C6		Small	25	288	96	0.48	
LKZA	D1	Believer		27	192	97	0.63	(Elk, 2015)
	D2	Sceptic		27	192	98	0.59	
GJUR	E1	Session 1		37	640	92	0.48	(Hedge et al., 2018)
	E2	Session 2		37	640	92	0.46	
TUPW	F1	Control	Pictures: Negative	40	240	96	0.45	(Kalanthroff, 2023)
	F2		Neutral	40	240	97	0.45	
	F3		None	38	240	97	0.43	
	F4	GAD	Negative	40	240	97	0.45	
	F5		Neutral	39	240	98	0.44	
	F6		None	38	240	98	0.42	
PBCZ	G1	DS		45	16		2.38	(Fontana et al., 2021)
	G2	Children		61	16		1.82	
VHLVb	H1	Children		27	48	90	1.20	(Morris et al., 2021)
	H2			62	48	94	1.04	
	H3			68	48	96	0.89	
FXLEa	I1	Session 1		59	96	95	0.36	Our replication of (Dale & Arnell, 2013)
	I2	Session 2		64	96	95	0.36	
QJPG	J			93	120	96	0.51	(Wong et al., 2021)
ZPLU	K			120	160		0.59	(Zappullo et al., 2023)
AYEZ	L			124	192	95	0.56	(Retzler & Retzler, 2023)
WLSQ	M1		None	189	600	92	0.58	(English et al., 2017)
	M2		Trained	187	600	91	0.52	
MBOC	N1		Pictures: Cute	233	84		0.56	(Álvarez-San Millán et al., 2022a)
	N2		Neutral	230	84		0.56	
	N3		Threatening	231	84		0.57	
LMOR	O			245	160	91	0.53	(Álvarez-San Millán et al., 2021)
MXMN	P			412	80	95	0.43	(Hayward et al., 2018)
LGYN	Q			900	320	94	0.49	(Gerlach & Poirel, 2018, 2020; Gerlach & Starrfelt, 2018)
BGIY	R			6099	96	97	0.84	(Duchaine et al., 2007a, 2007b; Germine et al., 2012)

Other elements of the task/procedure design (e.g., stimulus duration and position), figure design (e.g., size, density, shapes), experimental manipulations (e.g., induced mood), and participant demographics and clinical status (e.g., children, attention-deficit hyperactivity disorder (ADHD), autistic spectrum disorder (ASD), Down syndrome, anorexia) vary between the studies. One study (tag EZIG) is an ‘auditory Navon task’, an approach that we found interesting and wanted to present alongside the visual tasks. One dataset (WLSQ) is based on a study with an unconventional definition of congruence, where a trial counts as congruent when local and global information is of the same *type* (both are letters or both are numbers), rather than being exactly the same (e.g., both are the letter H).

It is important to keep in mind that our sample of studies is a non-systematic collection of different task designs (e.g., durations), figure designs, and participant characteristics. For the purpose of this study, we are interested in general, approximate patterns (correlations, reliabilities) of conceptually relevant effects/metrics and do not attempt to systematically interpret differences between task designs or participant groups.

All values are after trial and participant exclusion.

LGYN contains partially unpublished data, BGYI previously unpublished data.

*AN*, anorexia nervosa, *ASD*, autism spectrum disorder, *DS*, down syndrome, *GAD*, generalised anxiety disorder.

Dataset WLSQ consists of two experiments with the same participant demographic but different trial numbers. As trial numbers were very large in both cases (reported trial number (Tr) is the mean of both experiments), we deemed the difference between the two experiments negligible and merged them. See the cited studies for more details.

### Trial and participant exclusion

In trial data we removed all trials shorter than 200 ms or longer than 5,000 ms and then removed trials with reaction times 2.5 standard deviations beyond the participant’s and trial type’s mean. These exclusion criteria combine common criteria in our sample of studies (most commonly either a ≈200 – ≈2,000 ms range or a maximum of 2.5 standard deviations beyond the mean). To remain consistent with the common calculation method in the summary datasets, we summarised by taking the mean of correct trials. We then merged these directly calculated summaries with the datasets that were provided as summaries (summary level exclusions apply to all datasets). From these summaries, we excluded participants whose mean accuracy was lower than 0.6 or, in case of the auditory study (EZIG), 0.4, as its accuracy was overall much lower (see Table 1). We furthermore excluded participants whose mean reaction time was 2.5 standard deviations smaller or larger than the mean reaction time of their grouping (set × participant group × task condition). When summary data were reported only by the congruence × level combination, we calculated the mean level and mean congruence data as well (for OPS and OIS). We then calculated metrics for each participant, as shown in Fig. 2.

### Analyses, tests, significance

To test the *incongruent interference assumption*, we computed the proportion of negative LIS and GIS. To test the *interference independence assumption* and *unitary bias assumption*, we computed the Pearson correlation for GIS-LIS, BIS-SIS, and BIS-CPS, respectively. All results were entered into multi-level meta-analyses (Van den Noortgate et al., 2013) with the R-package *meta* (version 7.0.0).

All analyses are performed within grouping (set × group × condition) to avoid effects like Simpson’s paradox (Kievit et al., 2013). In the meta-analyses, we use clustering to account for the fact that groupings within a single dataset are not fully independent (same participants and/or same task design). Design similarities between different datasets are not accounted for. As the task design and participant characteristics are neither structurally nor empirically representative, there is no clear systematic way to weigh datasets and groupings. To avoid an over-representation of studies with many participants, the R-package *meta* weighs studies by their variance – more specifically, a dataset’s inverse variance divided by the sum of the inverse variance of the other datasets (Schwarzer et al., 2015). The very large dataset R, for example, contains more than half of all participants but is only weighted around 5% in most analyses. The specific weights of each grouping can be found in the meta-analysis forests plots (see Online Supplemental Material, Appendix #6).

We report the Bayes factor alongside the frequentist p-value, because whereas the p-value can only provide evidence *against* H0 (and thereby implicitly for H1), the Bayes factor can provide explicit evidence for and against H0 and H1. For p-values, we use the common significance-threshold of 0.05. For Bayes factors, we follow the interpretation that Bayes factors between 0.3 and 3 are uncertain, Bayes factors lower than 0.3 and 0.1 indicate moderate and strong evidence for H0, respectively, and Bayes factors higher than 3 and 10 indicate strong and very strong evidence for H1, respectively (Kass & Raftery, 1995). We use the R package *BayesFactor* to calculate the Bayes factor from participant data and use the functions provided in https://osf.io/cabmf/ (Wagenmakers et al., 2016) to calculate a Bayes factor from participant number and Pearson r for the significance-curves in our plots. For Bayes, we assume a uniform prior distribution in all tests. All significance tests (Bayes and frequentist) are two-sided.

## Results

### Overview

**Fig. 4:**
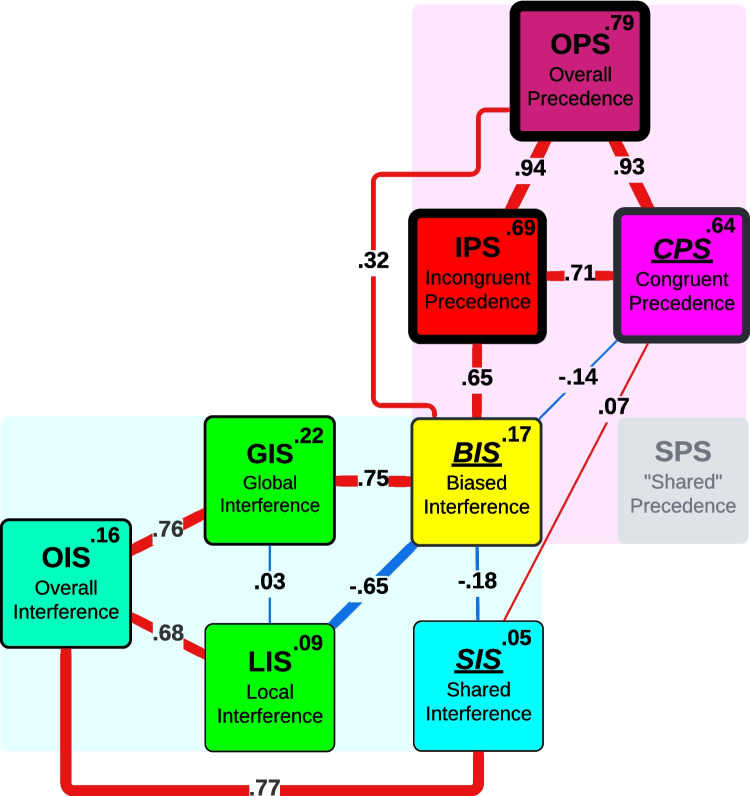
Reliabilities and important correlations of all metrics. The rim around metric-bubbles and the top-right number indicates metrics’ aggregate split-half reliability across all datasets that were provided as trial data. The connections between metric-bubbles indicate the strength of the aggregate correlation across all datasets (all meta-analyses in Online Supplemental Material, Appendix #6 or the project repository https://osf.io/gtqu8/). The bubbles in the bottom left are interference metrics, the bubbles in the top right are precedence metrics. BIS at the intersection is the interaction between both effect groups. The conceptually irrelevant SPS is only shown for structural completeness. The colours were chosen to represent proposed effect relations, with the three central effect-metrics BIS, CPS and SIS at the core (primary colours, underlined), GIS and LIS being a mix of BIS and SIS, and IPS being a mix of BIS and CPS

Figure 4 summarises the main empirical results, namely split-half reliabilities and between-metric correlations. Central findings are that interference metrics have a low split-half reliability, that the triangle of *conceptually* distinct effect-metrics – BIS, SIS, and CPS – are also *empirically* distinct (not correlated), and that GIS and LIS are empirically independent. Note that some correlations are near-trivial, like the correlation between OIS and GIS/LIS or between OPS and CPS/IPS, because the overall metrics are half consistent of the trials that make up their more specific sub-metrics (compare Fig. 2). Note also that low split-half reliabilities have implications for between-metric-correlations. For specific interpretations see the following sections that discuss the reliabilities and correlations within the conceptual framework outlaid before.

### Split-half reliability

**Fig. 5:**
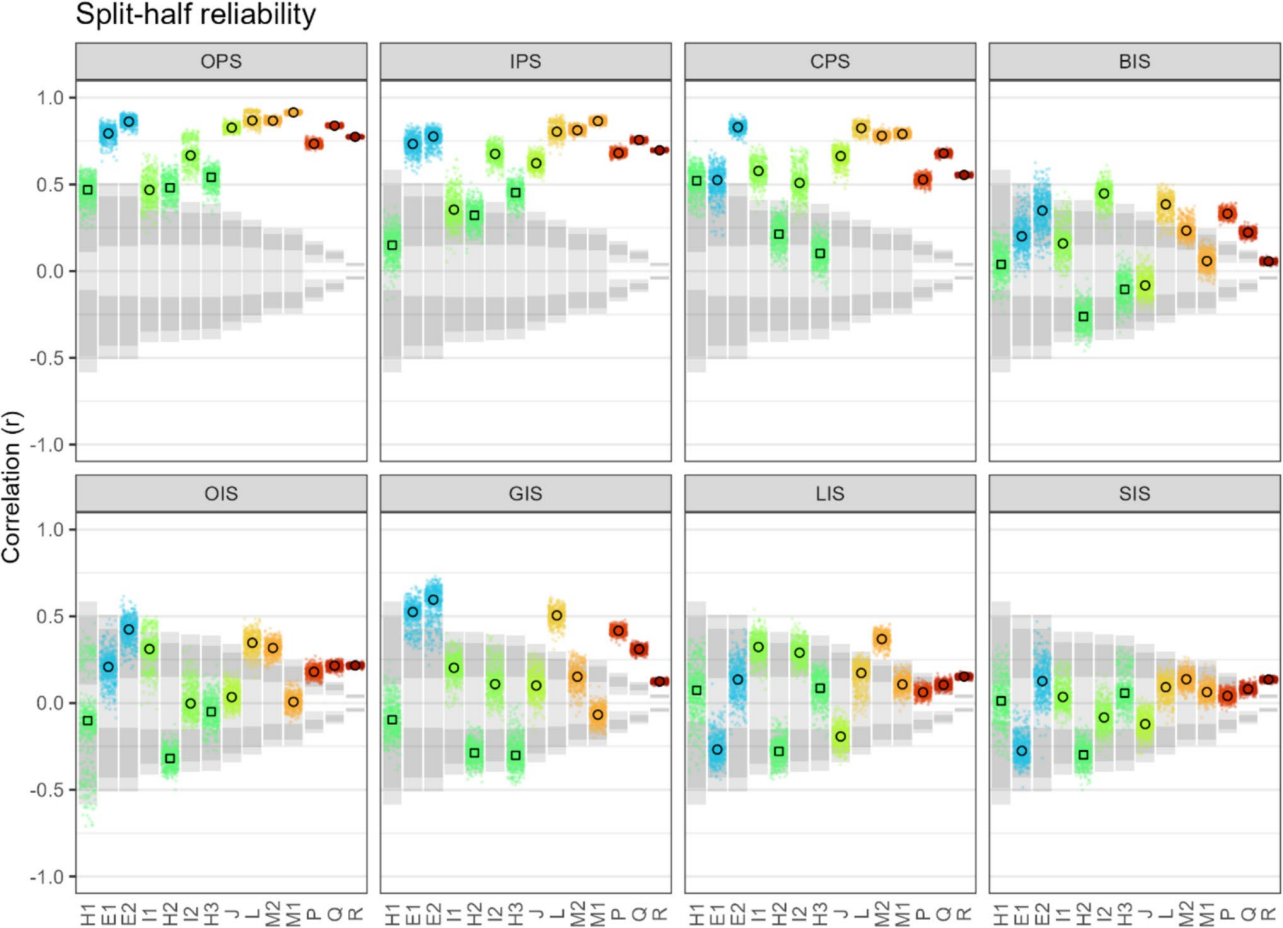
Split-half reliability of metrics in each dataset for which trial-data is available. The small dots represent 500 bootstrapped Pearson correlations, each with 80% of the grouping’s participants, the larger dots their median. The shaded bars indicate the negative and positive ranges of Pearson r which – for each grouping’s participant number – correspond to Bayes factors between 0.1 and 0.3 (inner light shade, moderate evidence for non-correlation), 0.3 and 3 (dark shade, uncertain evidence), and 3 and 10 (outer light shade, moderate evidence for correlation). Overall, congruent and incongruent precedence score have a moderate to strong split-half reliability and all metrics involving interference have a very low to moderate split-half reliability. CPS, IPS, GIS, LIS and SIS are each based on half the trials. BIS is technically based on all trials, but only through taking the difference between IPS and CPS (or GIS and LIS). Trials were split in half by grouping trials by level and congruence and then alternatingly assigning them to half A or B. The two precedence metrics CPS and IPS have a moderate to strong split-half reliability, while the two interference metrics GIS and LIS and the two ‘second order’ metrics BIS and SIS have a very weak to moderate split-half reliability. To calculate split-half metrics in those datasets that provide trial-data, we grouped each participant’s trials by Level and Congruence and alternately assigned them to half 1 or half 2

A measure for the split-half reliability is the Pearson correlation of a metric between two halves of a participant’s data (such as odd vs. even trials). Interference metrics show a concerningly low reliability, in particular LIS (0.09, 95%CI [−0.01; 0.20]) and SIS (0.05, 95%CI [−0.01; 0.10]), but also GIS (0.22, 95%CI [0.05; 0.39]), OIS, and BIS (0.17, 95%CI [0.05; 0.29]) (Fig. 5, meta analyses in Figs. 13, 14 and 15 in Online Supplemental Material, Appendix #6). Removing participants with (conceptually impossible) negative scores in GIS, LIS, and SIS increases the aggregate reliability of all three metrics by about 0.1, which is still low (Fig. 21, meta analyses in Figs. 13, 14 and 15 in Online Supplemental Material, Appendix #6). Such low split-half reliabilities for Navon-effects have been reported previously (Gerlach & Poirel, 2018, 2020; Hedge et al., 2018), as well as that LIS has a an even lower reliability than GIS.

Furthermore, it should be noted that these low aggregate reliabilities are in part driven by *negative* correlations in datasets with fewer participants and – in the case of H1–H3 – also with relatively few trials. As examined in more detail before (Hedge et al., 2018), reliability in the Navon task increases somewhat with larger trial numbers before reaching a plateau in the low triple-digits (reliability vs. trial number in Fig. 22 in Online Supplemental Material, Appendix #7).

Interestingly, the reliability is higher for GIS than LIS in some groupings and the other way around in others. While this may be trivial noise, there seems to be a pattern, namely that datasets using figures who employ relatively large and sparse local elements (such as in Fig. 1) show a higher LIS reliability (e.g., I, M) and vice versa (e.g., L). Thus, the figure design might strengthen either local-to-global interference and weaken global-to-local interference or vice versa. Taking the simple reliability-aggregate over both types of figures may therefore underestimate real reliability that GIS and LIS *can* have depending on the task design. These results should thus be generalised with caution to results for GIS and LIS in the literature. The relative reliability of GIS and LIS may also serve as a window to a more mechanistic understanding of the interference effect, in particular with specifically designed experiments.

Besides its inherent meaning, the reliability should also be considered as a confounding factor when interpreting the correlation of two metrics. When a metric has a low reliability, its correlations are more likely to be weak, as the metric’s main effect is either masked by other effects or random noise. Statistically correcting for low reliabilities is tricky and can lead to values that are very difficult to interpret. We therefore do not reliability correct the correlations in this manner in this paper (see details in Online Supplemental Material, Appendix #7).

### Incongruent interference assumption

The *incongruent interference assumption* predicts that there should – in principle – be no negative LIS and GIS, as incongruent trials should take longer than congruent trials due to cross-level interference. However, we find that nearly all studies have participants with negative LIS and GIS, with an overall proportion of 0.23 (95%CI [0.19; 0.27]) negative LIS and 0.10 (95%CI [0.06; 0.16]) negative GIS according to our meta-analysis (see Online Supplemental Material, Appendix #6, Fig. 16 and 17). The distribution of LIS tends to be closer to 0 than the distribution of GIS (Fig. 30 in Online Supplemental Material, Appendix #9) and thus if noise is assumed to be equal for both, a larger share of negative LIS can indeed be expected.

It is important to note that distributions of GIS and LIS overlap in some datasets and in others the relationship is even reversed with GIS closer to 0 than LIS (Fig. 30 in Online Supplemental Material, Appendix #9). Visual comparison of the stimuli used in the corresponding experiments with the stimuli used in the other experiments suggests that GIS and LIS might have equal distributions when the local elements are comparatively few, sparse, and large. Indeed, the stimuli we used in our replication-experiment (dataset I) had been designed to achieve a balance between local and global bias in the population (Dale & Arnell, 2013).

**Fig. 6:**
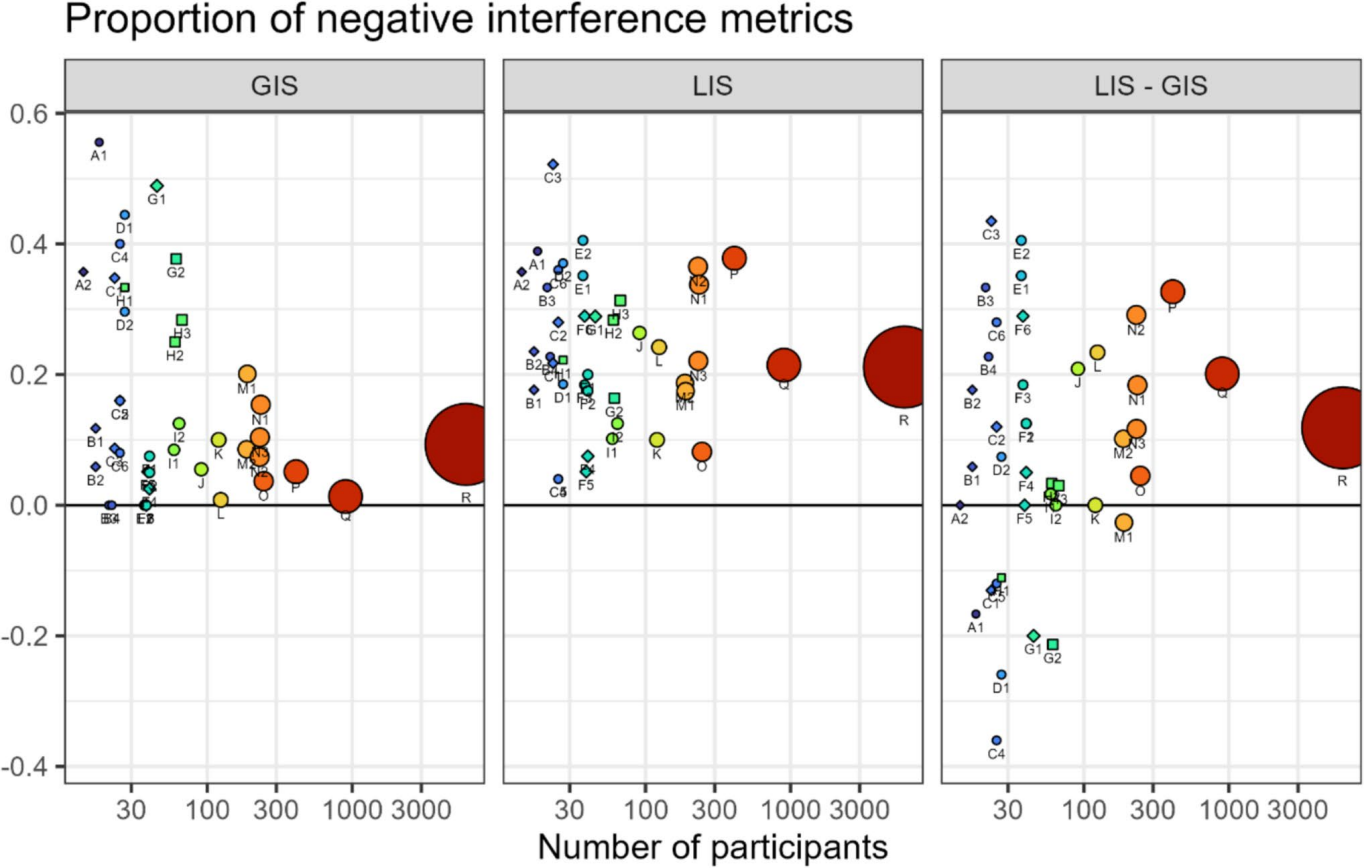
Proportion of participants with negative GIS and LIS, plotted against the number of participants (also represented by the area of the points). Children are shown as squares, clinical populations as diamonds, neurotypical adolescents and adults as circles (see Table 1). LIS tends to have higher proportions of negative values than GIS (difference shown in right panel), although the proportions of negative values is high in some of the smaller datasets

The magnitude and direction of the difference between the distribution of GIS and LIS (and proportions of negative scores) is thus design related and more of *theoretical* interest. *Conceptually* relevant remains the *empirical* existence of any negative scores at all that cannot be explained based on the *incongruent interference assumption* alone. *Empirically*, a certain proportion of negative scores can be expected given that all positive values down to 0 are conceptually valid and empirical sampling error can produce a spread beyond the conceptually expected (Haaf & Rouder, 2019). However, it is difficult to determine whether the proportions of negative interference scores that we observe can still be considered random effects and measurement errors or whether they hint at a conceptually significant latent effect (which would be congruent interference, see Assumption 1 in the *Assumptions* section). A potential approach is to assume that distribution spread caused by noise is similar in GIS and LIS to infer that LIS must contain *opposing* effects that cancel each other out but are taken together of a similar magnitude as GIS, to explain why the spread of GIS and LIS is so similar (see details and data in Online Supplemental Material, Appendix #9, Fig. 30 and 31). However, this line of reasoning is highly speculative. Without task variations that were specifically designed for this question, we expect it to be difficult to gain proper empirical evidence for or against the *incongruent interference assumption* (Fig. 6).

### Precedence-interference independence assumption

Assuming then that the *incongruent interference assumption* holds, and the only cross-level effect is interference in incongruent trials, congruent trials contain the biased precedence effect and incongruent trials contain the biased precedence effect as well as the biased interference effect. Since no trials without biased precedence exist, interference effect sizes need to be calculated from the total effect and the biased precedence effect. This requires a further assumption on how these two effects synergise.

As outlined in the *Assumptions* section, we follow the common assumption in the literature that the effects are rigid and do not synergise. However, this assumption cannot be examined conceptually and is difficult to examine theoretically and empirically. Nevertheless, our meta-analysis might give us a hint on the synergy between the effects.

Namely, we correlate the biased precedence effect (CPS) and biased interaction effect (BIS). Note that in principle, this correlation can contain any number of theoretically relevant interactions/synergies between the two effects. We therefore come back to this same correlation for our empirical analysis of Assumption 4 later, but the direction of the correlation makes it relevant for Assumption 2 as well. Namely, we find a very weak *negative* correlation between CPS and BIS (−0.14 (95% CI [−0.22; −0.05]), see Fig. 9 and Online Supplemental Material, Appendix #6, Fig. 20).

Theoretically speaking, we would not expect that a global biased precedence is linked with local biased interference and vice versa. Assuming that this negative correlation does not represent a legitimate relationship, we can infer that it is caused by a ‘soft’, gradual ceiling effect where large global CPS and large global BIS do not add up linearly but rather a certain amount of global bias is ‘lost’ in the observation due to this soft ceiling effect.

However, this is a highly speculative interpretation and should be treated carefully. Without knowledge about the local–global bias mechanisms and the exact causes for precedence and interference biases, every interpretation remains hypothetical. just like with the *incongruent interference assumption*, we therefore assume that the *precedence-interference independence assumption* holds, in order to proceed with our examination of the other assumptions.

### Interference independence assumption

**Fig. 7:**
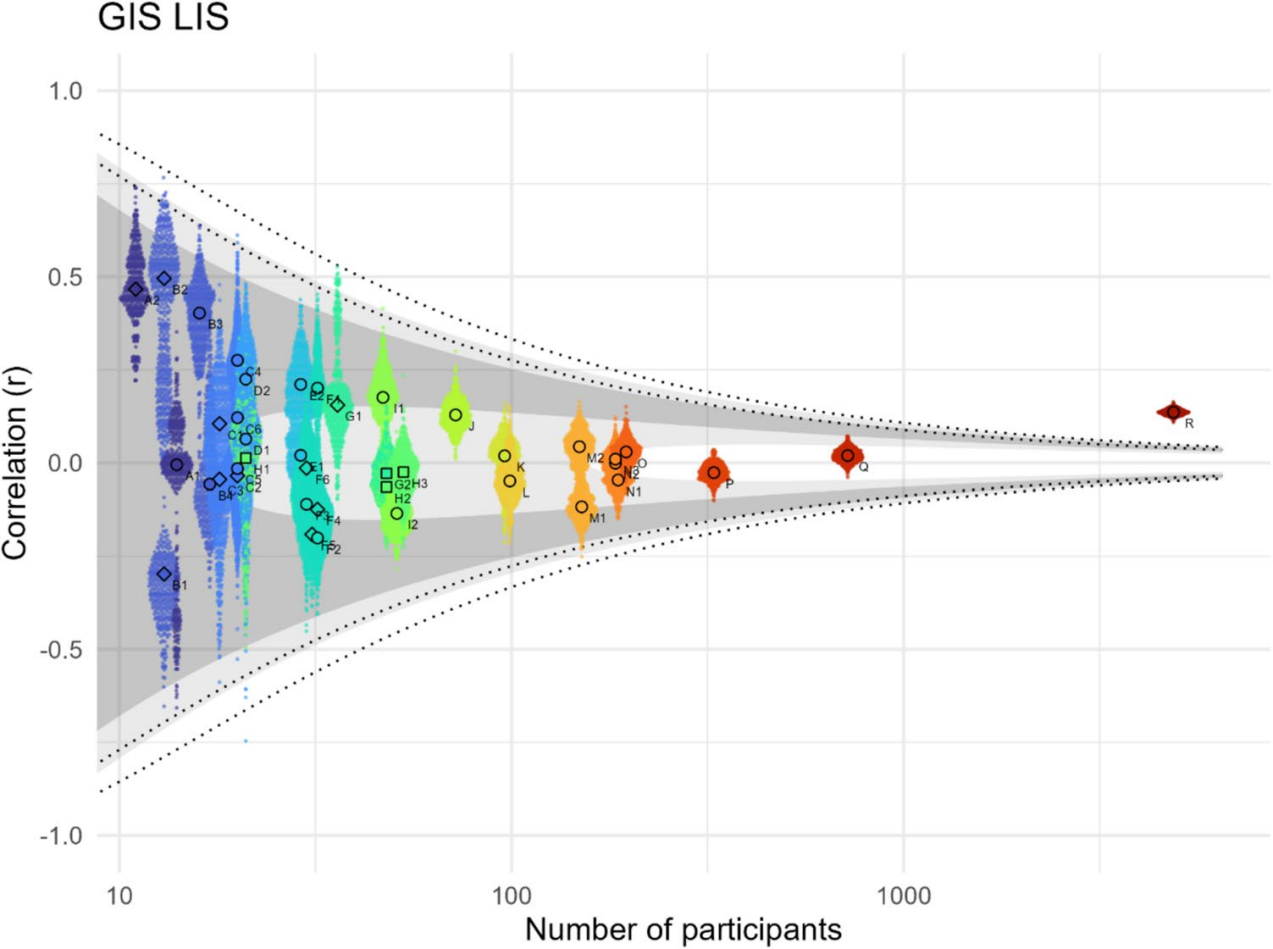
Pearson correlations between GIS and LIS across different groupings. The small dots represent 500 bootstrapped Pearson correlations, each with 80% of the grouping’s participants, the larger dots their median. The shaded area indicates the negative and positive range of Pearson r which – for a given participant number – correspond to Bayes factors between 0.1 and 0.3 (inner light shade, moderate evidence for non-correlation), 0.3 and 3 (dark shade, uncertain evidence), and 3 and 10 (outer light shade, moderate evidence for correlation). The inner (outer) dotted lines show Pearson r corresponding to a p-value of 0.05 (0.005). Children are shown as squares, clinical populations as diamonds, neurotypical adolescents and adults as circles (see Table 1)

The *(global–local) interference independence assumption* predicts that LIS and GIS are not correlated and we find indeed that the correlation is only significant in the largest dataset (S), and in most other datasets the Bayes factor shows moderate or even strong evidence for H0 (Fig. 7). The effect strength over all studies is 0.03 (95% CI [−0.01; 0.07]) according to our meta-analysis (see Online Supplemental Material, Appendix #6, Fig. 18). GIS and LIS can thus be seen as behaviourally independent measures, but two important caveats apply:

Firstly, this empirical result does not strictly imply that GIS and LIS describe independent *theoretical* constructs or mechanisms, as we showed before (see *interference independence assumption* in the *Assumptions* section). And secondly, the empirical non-correlation of LIS and GIS is at least in part driven by the low split-half reliability of both metrics (Fig. 5). This means that the metrics capture a weak main effect masked by random effects and due to this noise, it is difficult to determine whether the observed non-correlation is driven only by the noise or by the noise plus a latent non-correlation of the weak main effects. As outlined in Online Supplemental Material, Appendix #7, quantifying the influence of low reliabilities is tricky.

### Alternative interference interpretation

We also want to briefly present an alternative way of looking at interference independence, namely by splitting GIS and LIS (which are non-biased, mixed-effect interference metrics) into BIS and SIS (which are the single-effect metrics).^2^ Note that BIS is a biased effect, but GIS, LIS and SIS are not. By correlating BIS and SIS we can assess whether participants with a stronger non-biased (‘baseline’) interference in both global and local trials (larger SIS) are more likely to show an additional local or an additional global interference bias (negative or positive BIS).

**Fig. 8:**
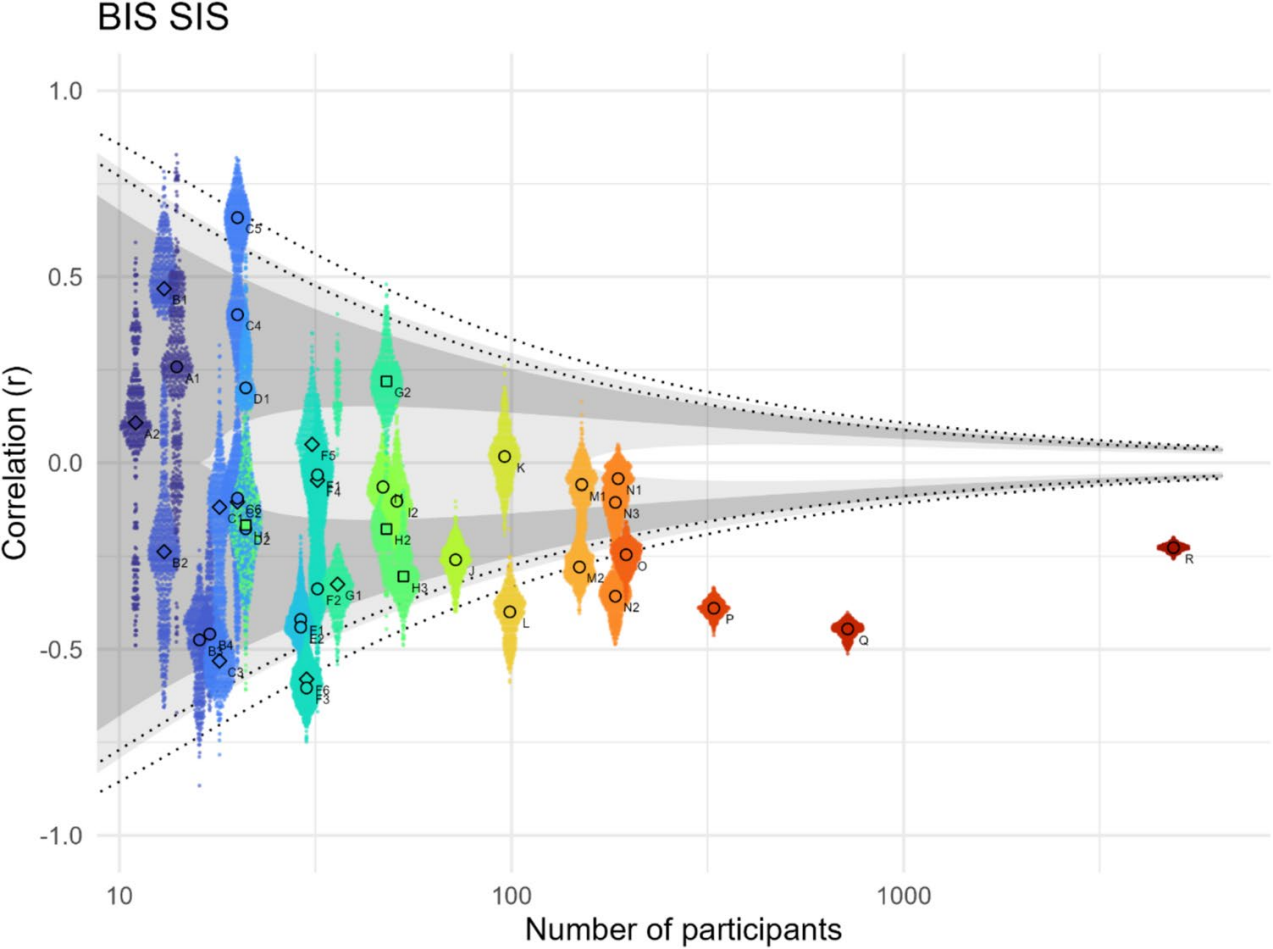
Pearson correlations between BIS and SIS across different studies. The small dots represent 500 bootstrapped Pearson correlations, each with 80% of the grouping’s participants, the larger dots their median. The shaded area indicates the negative and positive range of Pearson’s r which – for a given participant number – correspond to Bayes factors between 0.1 and 0.3 (inner light shade, moderate evidence for non-correlation), 0.3 and 3 (dark shade, uncertain evidence), and 3 and 10 (outer light shade, moderate evidence for correlation). The inner (outer) dotted lines show Pearson’s r corresponding to a p-value of 0.05 (0.005). Children are shown as squares, clinical populations as diamonds, neurotypical adolescents and adults as circles (see Table 1)

The correlations show mixed results, with some, especially larger datasets, showing moderate to strong evidence in favour of a negative correlation and other datasets showing uncertainty or even moderate evidence for H0 (Fig. 8). The effect strength of this correlation over all studies is −0.18 (95% CI [−0.26; −0.10]) according to our meta-analysis (see Online Supplemental Material, Appendix #6, Fig. 19). In some datasets, participants with a stronger interference effect in local and global trials (SIS) have a small tendency to show an excess amount of local-to-global interference (negative BIS). The level-neutral and level-specific interference effects are thus nearly, but not fully independent, and it seems that a higher level-independent proneness to interference is linked with a stronger local-to-global interference.

Again, the low split-half reliabilities (weak to moderate for BIS and very weak for SIS, Fig. 5) must be considered in this correlation. Furthermore, SIS has the issue of negative values that are conceptually impossible and thus tricky to process. Excluding negative interference scores does not alter the pattern of correlations (Fig. 29, Online Supplemental Material, Appendix #8).

While BIS and SIS are *conceptually* independent and to our knowledge no *theoretical* link between the two has been discussed so far, the *empirical* evidence points towards a potential relationship between the two that could be of theoretical significance.

### Unitary bias assumption

**Fig. 9:**
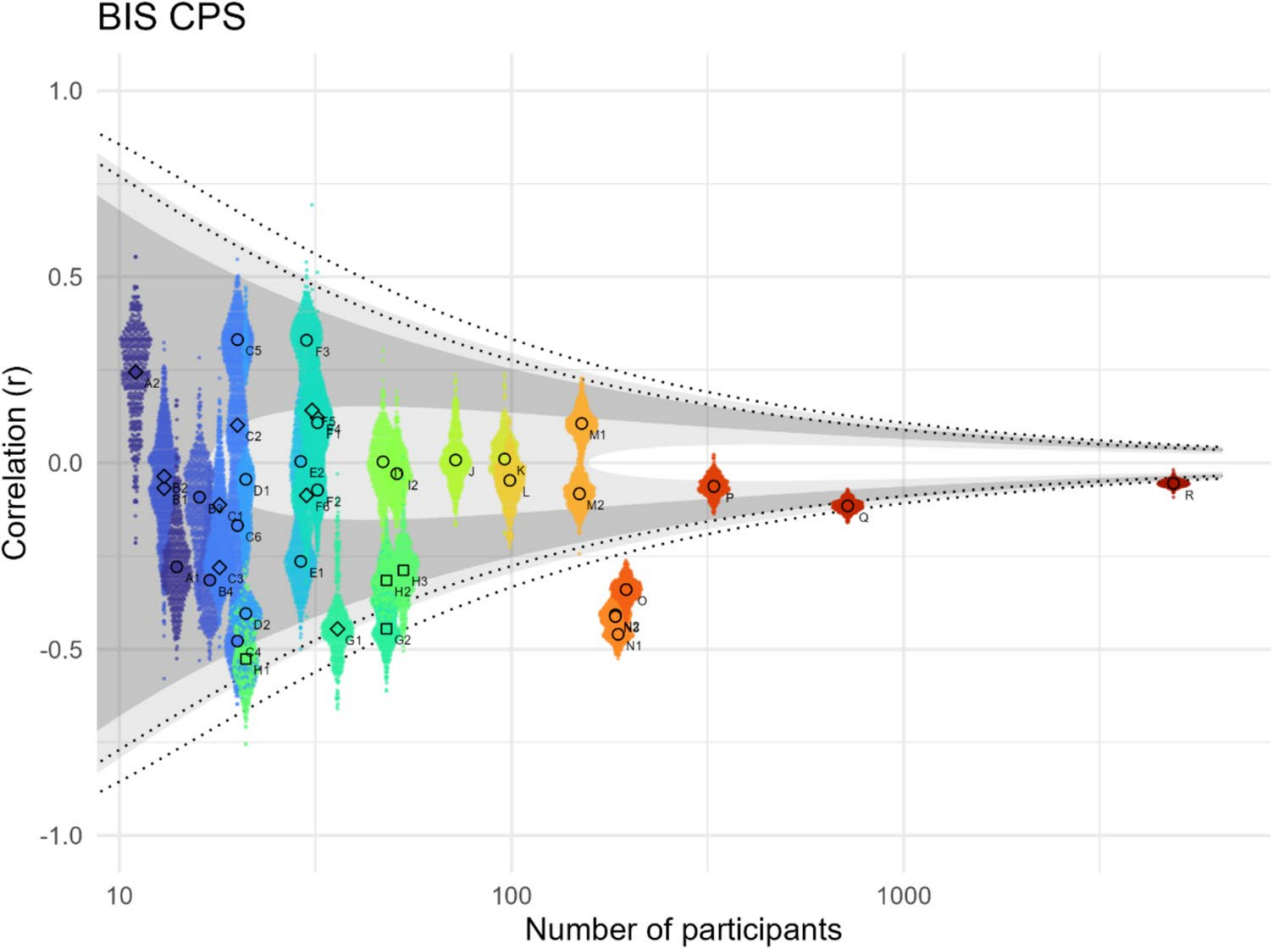
Pearson correlations between BIS and CPS across different studies. The small dots represent 500 bootstrapped Pearson correlations, each with 80% of the grouping’s participants, the larger dots their median. The shaded area indicates the negative and positive range of Pearson’s r, which – for a given participant number – correspond to Bayes factors between 0.1 and 0.3 (inner light shade, moderate evidence for non-correlation), 0.3 and 3 (dark shade, uncertain evidence), and 3 and 10 (outer light shade, moderate evidence for correlation). The inner (outer) dotted lines show Pearson’s r corresponding to a p-value of 0.05 (0.005). Children are shown as squares, clinical populations as diamonds, neurotypical adolescents and adults as circles (see Table 1)

The *unitary bias assumption* predicts that CPS and BIS are representative of a single bias and therefore positively correlated, but we do not find empirical evidence for such a correlation (Fig. 9). In fact, the effect strength over all studies is even slightly negative with −0.14 (95% CI [−0.22; −0.05]) according to our meta-analysis (see Online Supplemental Material, Appendix #6, Fig. 20). Interpreting a negative BIS-CPS correlation is conceptually tricky (see *Precedence-interference independence assumption* in the *Assumptions* section) and due to the weakness of the effect we will not do so, but want to mention that a ‘soft’ (gradual) ceiling effect between the compounding interference bias and congruence bias could play a role. In other words, large effect sizes in the same bias-direction (e.g., both global) might not stack linearly in incongruent trials (CPS + BIS = IPS) but rather become less than the sum of the two (e.g., CPS + 0.9 × BIS = IPS), leading to a negative correlation between CPS and BIS.

The split half-reliability of BIS is quite low, but the split-half reliability of CPS is moderate to strong (Fig. 5). To put the reliabilities of these metrics into perspective, one can compare the correlation between them. IPS and BIS (Fig. 25 in Online Supplemental Material, Appendix #8) are strongly correlated despite the low reliability of BIS. The correlation between IPS and CPS is only slightly weaker (Fig. 26 in Online Supplemental Material, Appendix #8). These correlations likely reflect that conceptually speaking, the biased precedence effect exists in both IPS and CPS, lending credibility to the empirical non-correlation of BIS and CPS.

As there is no evidence for a *positive* correlation between BIS and CPS, we can state fairly confidently that the *unitary bias assumption* does not hold and that the two conceptually distinct effects of biased precedence (CPS) and biased interference (BIS) are empirically independent, although with the caveat of the low reliability of BIS. According to this analysis, a full representation of the local–global bias as captured by the Navon task (and likely other implementations of the local–global paradigm) therefore necessarily includes both, CPS and BIS.

## Discussion

### Summary

In this paper we specified the possible metrics that can be calculated from the Navon task. These metrics (besides the newly introduced SIS) are currently used in the literature in an inconsistent manner. Through a conceptual analysis we then demonstrated that the metrics CPS and BIS capture the biased precedence and biased interference, respectively, and that the metric IPS captures a mixture of these two effects. An empirical correlation can show whether two conceptually distinct effects are empirically independent as well, as was done previously, for example for GIS and CPS (Gerlach & Poirel, 2018, 2020). Across over a dozen datasets we find that the conceptually valid bias metrics CPS and BIS are not correlated. Therefore, the assumption of local–global bias as a unitary construct (4) does not even hold within a singular task design like the Navon task. This finding has implications for all research that uses a singular bias score, which includes a large number of studies that neglect congruent trials altogether and use only the IPS.

For LIS and GIS, it is not clear whether they capture theoretically relevant effects or whether they should be viewed as a mixture of the effects captured by BIS and SIS. We show that empirically GIS and LIS are independent, and that therefore – empirically – the *interference independence assumption (3)* holds, but the theoretical significance and validity of these two metrics is not clear without a mechanistic understanding of cross-level interference. As GIS and LIS in themselves do not represent a bias, we argue that the BIS should be used as a representation of the biased interference effect.

Importantly, the calculation of all metrics relies on two more assumptions of fundamental theoretical nature. The first of these assumptions is that the only cross-level effect present in the Navon task is an interference in incongruent trials (1) and the second is that precedence and interference effects do not synergise in incongruent trials (2). The *precedence-interference independence assumption (2)* is hardly empirically testable due to circular logic, as the calculation of the metrics (CPS and BIS) that would be used to test it, is based on the assumption itself. Furthermore, whether a particular interaction (or correlation) between CPS and BIS is an artifact of the measurement/calculation or an expression of the true relation between the basic precedence (CPS) and biased interference effects (BIS) seems very difficult to disentangle. The weak empirical trend towards a *negative* correlation between CPS and BIS potentially hints towards a ‘soft’ ceiling effect of the reaction times, which would partially undermine the validity of *observable* BIS, but not necessarily its theoretical significance.

The *incongruent interference assumption (1)* predicts strictly positive GIS and LIS, which does not hold empirically (Fig. 6). If the negative interference scores reflect more than noise (which is not clear), then these scores can only be explained by the (theoretically unlikely) incongruent facilitation or the (theoretically likely) congruent interference effect, which would overcompensate the positive interference scores from the (theoretically likely) incongruent interference and (theoretically possible) congruent facilitation effects. An attempt at an exhaustive analysis of the *incongruent interference assumption* (i.e., the four possible cross-level influences, Fig. 3) is beyond the scope of this paper, but is in our eyes important for a better understanding of the task.

Beyond the implications for the Navon task, the structured analysis of task factors we present is not strictly limited to local–global or hierarchical figure tasks and should *generally* apply to all tasks with a combination of congruency and level, i.e., tasks with sometimes congruent and sometimes incongruent information on two or more levels and where only one of these levels contains relevant information. Examples for such tasks are the Stroop task (MacLeod, 1991; Stroop, 1935) or the picture-word interference task (van Maanen et al., 2009). Our approach to the structured analysis of cognitive tasks may even serve as inspiration for the analysis of more dissimilar multi-factor cognitive tasks.

### Theoretical complexities

While our analysis has a conceptual and to a smaller degree an empirical focus, we also want to highlight three theoretical aspects of the local–global paradigm that underline its complexity.

Firstly, the *interference*. The usual interference-design in local–global tasks contrasts incongruent trials with congruent trials, akin to interference in the Stroop task. Congruent and incongruent trials are presented randomly and information on both levels varies randomly across trials as well. However, an alternative interference paradigm exists, namely contrasting a block of ‘unpredictable’ incongruent trials (i.e. classical incongruent trials) with a block of ‘predictable’ incongruent trials, in which the non-target level information is the same for every trial (local trials: fixed global shape).

This so-called Garner-interference (Garner, 1969) has its own particular conceptual interpretation (Niv et al., 2022) and importantly was found to be not correlated with the Stroop-like interference paradigm (Pomerantz et al., 1994) that is commonly used in local–global tasks and the Navon task specifically. This suggests that two different interference constructs (Stroop and Garner) might exist in the local–global paradigm as well, adding even more complexity.

While Garner interference and Stroop interference may differ on the theoretical level, they are identical from a conceptual perspective and therefore the metric-framework presented in Fig. 2 applies to both of them. As the two interference-paradigms are based on different task designs, empirical analysis of Garner-interference is not possible with our data.

Secondly, the *stimulus.* Hierarchical compound figures are usually rather sterile, and it is not clear how their effects translate to more natural visual stimuli and stimuli that lead to proper Gestalts. In proper Gestalts, local elements tend to play a more significant role in the emergence of the Gestalt than they do in hierarchical compound figures, where a single local element is a relatively insignificant part of the global ‘Gestalt’ (if at all). While local–global research has emerged from Gestalt research (de-Wit & Wagemans, 2014), it might have effectively diverged from it through the use of the now typical hierarchical compound figures.^3^

However, in regard to natural stimuli it should be mentioned that a global bias seems to be linked to the holistic processing of faces and natural stimuli (Gerlach & Poirel, 2018; Gerlach & Starrfelt, 2018; Gerlach et al., 2017), suggesting that local–global effects may result from more general perception mechanisms.

In regard to Gestalt research, it is interesting to note that Navon’s work fits the information-processing approach better than the Gestalt approach while Asch’s original approach to hierarchical compound figures (Asch, 1962) was closer to the Gestalt approach (Ash was a long-time friend of Max Wertheimer, the founder of the Berlin school of Gestalt psychology). For further discussion of proper Gestalts in perceptual organization, see Wagemans (2018), Wagemans et al. (2012a), and Wagemans, Feldman and et al., (2012a, 2012b).

Thirdly, the *broader generalizability*. Many psychological tasks have some form of local and global level, although usually less explicit than in the Navon task. The notion of a single, overarching conceptual style (Förster & Dannenberg, 2010) within an individual or a group implies not only that the biased precedence and biased interference should be correlated (which is not the case, Fig. 9), but also that there is some consistency across different task designs. Stronger or weaker forms of this notion have been quite pervasive in the local–global literature – at least implicitly. However, only few papers empirically investigate the link between different implementations of the local–global paradigm as well as the Navon task and tasks outside the local–global paradigm. These papers did not find clear correlations between different implementations of the local–global paradigm (Dale & Arnell, 2013; Lacko et al., 2023) or local–global tasks and the embedded figures task (Booth, 2006; Chamberlain et al., 2017), suggesting that many effects are at play.

### Interpretation and implications

These theoretical points lead to the profoundly important question how many different aspects there are to the deceptively very simple concept of a bias towards information on a local or global level. Our conceptual analysis and our empirical results demonstrate that the local–global bias effects of the Navon task stem from a multifaceted, design-dependent, and partially non-robust construct**.** Specifically, two conceptually and empirically distinct and valid bias metrics (CPS, BIS) should be considered when using the Navon task.

Moreover, the conceptual aspects of our analysis are not limited to the Navon task and apply in principle to all implementations of the local–global paradigm and probably to some degree to other tasks with a congruence-like task-factor and a level-like task-factor (such as the Stroop task). However, design-specific conceptual implications must be considered, and theoretical implications may differ (e.g., for divided-attention designs).

It is furthermore very important to stress that our paper has a conceptual and empirical focus and barely touches on the theoretical and mechanistic layer of the local–global paradigm. Questions such as whether the common hierarchical figures are suitable to examine part-whole perception and what perceptual mechanisms they are tapping into are not answered in this article. However, this theoretical layer is crucial for the relevance of the local–global paradigm as a whole and should always be considered.

Due to its conceptual-empirical focus, this article also does not intend to dispute any specific empirical findings or theoretical interpretations reported in the literature. While our conceptual analysis of a metric *might* have implications for the interpretation of a study using that metric, the theoretical validity and relevance of the reported effects does not *necessarily* suffer: OPS for example is strongly correlated with CPS (Fig. 27, Online Supplemental Material, Appendix #8) and a study that uses the *conceptually speaking* suboptimal OPS therefore likely reports a similar finding as the conceptually more specific CPS would have shown; studies that do not directly report BIS, but interpret differences between GIS and LIS in their discussion likely do report the relevant patterns of biased interference; etc. The conceptual (and theoretical) merit of each reported finding therefore has to be interpreted on a case-by-case basis.

Our main concern for local–global research is that a significant part of the literature puts insufficient focus on conceptual completeness and transparency. It thereby tends to underestimate the conceptual and theoretical complexity of the local–global paradigm. We see this as a major reason why – after decades of research – our theoretical understanding of *local–global bias* is still very limited and our knowledge about the paradigm is largely limited to empirical patterns of behaviour. Conceptual rigor in this kind of analysis or alternative analysis approaches like cognitive models (Turner et al., 2017) is in our opinion a powerful and necessary part of a true insight into the cognition of local–global bias. Such an insight will allow to integrate the construct with other cognitive constructs, bring the cognitive research closer to neuroscientific research, help with understanding the origin and implications of local–global biases of different clinical groups, and contribute to our understanding of the nuances of perceptual organization.

## Supplementary Information

Below is the link to the electronic supplementary material.

Supplementary file1 (DOCX 3738 kb)

## Data Availability

Formatted data is available in the data repository (the local–global Repo, https://osf.io/xtrhe/) as well as in the project repository (https://osf.io/gtqu8/).

## Footnotes

1 Please note that we use the term *synergy* instead of the term *interaction* to avoid confusion with the statistical interaction of the task factors Congruence and Level.

2 While the SIS-BIS independence does not relate to the GIS-LIS independence directly (in fact, GIS and LIS could *hypothetically* correlate strongly without determining the BIS-SIS correlation), we found it relevant to mention this correlation here as well, since it is a kind of interference independence.

3 One of the authors (Johan Wagemans) has been working on the construction of proper Gestalt-like compound stimuli, with some ecological validity. As this is a work-in-progress, we cannot refer to this in our present paper.

## Code availability

The code is available in the project repository (https://osf.io/gtqu8/).

## Declarations

### Open practices statement

Raw data, analysis code, and analysis-output are available in the project repository (https://osf.io/gtqu8/). Data are also available at the LoGloRepo (local–global repository, https://osf.io/xtrhe/). All dataset except our own (FXLEa) were obtained from existing studies, either from public repositories, supplementary material, or by contacting researchers. The analysis was not preregistered.
