# Supplementary material for: Understanding Navon: A detailed structural and conceptual analysis of a basic local–global task
Source: Psychon Bull Rev. 2025 Oct 20;32(6):3323–45. doi: 10.3758/s13423-025-02741-2 (PMC12627199; doi:10.3758/s13423-025-02741-2)
Supplement: Supplementary file 1 — Supplementary file1 (DOCX 3738 kb) [file 13423_2025_2741_MOESM1_ESM.docx]

# Appendix

**Understanding Navon: A Detailed Structural and Conceptual Analysis of a Basic Local-Global Task**

Felix Schweigkofler, Sjoerd Stuit, Johan Wagemans, Tanja Nijboer, Leendert van Maanen, Stefan van der Stigchel

## #1 Layers of abstraction

Each layer of abstraction that we use in this paper (structural, conceptual, empirical, theoretical) covers a certain aspect of the topic and has its own fundamental logic. We find that clearly differentiating between the layers in one’s thinking helps with clarifying and structuring conflicting ideas and interpretations.

**Structural layer**: Until now, while we did describe the conceptual meaning of the metrics, we classified them in a structural-statistical framework: Two task factors (conceptually speaking: Level, Congruence) with two instances each (local and global, congruent and incongruent) give rise to two main effects (OPS, OIS), four simple main effects (CPS, IPS, GIS, LIS) and an interaction effect (BIS) plus a non-standard effect (conceptually: SIS). This structural framework is a general mathematical description of effects for any two-by-two factors imaginable and contains no inherent *conceptual* meaning.

**Conceptual layer**: The conceptual meaning and (content) validity of any of these structural effects (the metrics) only arise from pushing conceptually meaningful data through this calculation-framework – in our case the summary statistics of the trial types. Analysing the conceptual meaning of the metrics (structural-statistical effects) of trial-type summaries of the Navon task – as we did above – shows that there are only three conceptually valid metrics: CPS, SIS, BIS.

Note that these metrics do not show a perfect symmetry within the structural framework, because the task factors are not conceptually symmetrical – structurally speaking, CPS is a simple main effect, BIS is an interaction effect, and SIS is outside of the structural framework. The CPS and BIS contain an influence of level and are therefore bias-metrics (since bias in this article always refers to local-global bias), while SIS explicitly only contains the effect of Congruence and is therefore a strict interference metrics. In Appendix #3 we visualize how these effects (except SPS) relate to each other by showing hypothetical participants with a range of different effect strengths and directions.

**Theoretical and empirical layers**: Critically, conceptual meaning does not automatically translate to theoretical meaning and empirical validity: A metric can be conceptually meaningful, but simply not exist as empirical effect, either in general or in a certain condition or certain demographics. Split-half reliability (see Figure 5) is one way of establishing an effect’s empirical validity. Also, just because a metric has a clear conceptual meaning, the described effect is not necessarily theoretically meaningful or useful. For example, BIS and SIS have clear conceptual interpretations, but they rest on the assumption that the difference between the global-to-local and local-to-global interference effects is theoretically meaningful, i.e. that it describes a relevant aspect of cognition.

A further potential layer is a mechanistic layer, i.e. an accurate description of the processing. For this paper, the four layers structural, conceptual, theoretical, and empirical should be sufficient, but in other studies other layers of thinking might become relevant to differentiate.

## #2 Score calculation example

In Figure 10 we show an example calculation of metrics. The grey bars represent the median reaction time in the condition in 100 ms, namely incongruent (I) and congruent (C), local (L) and global (G), as well as a combination of these (IL, IG, CL, CG). The coloured bars on top represent the score which results from the subtraction of the two bars used in the calculation, represented as dotted horizontal line. All subtractions are visualized such that the right bar is subtracted from the left bar. The calculations are the following:

- OIS = incongruent – congruent
- OPS = local – global
- IPS = incongruent local – incongruent global
- GIS = incongruent local – congruent local
- CPS = congruent local – congruent global
- LIS = incongruent global – congruent
- SIS = min{GIS,LIS} (in this case GIS)
- BIS = GIS – LIS = IPS – CPS

Positive values indicate a global bias (global target increases performance) and a “positive” interference effect (interference slows performance).


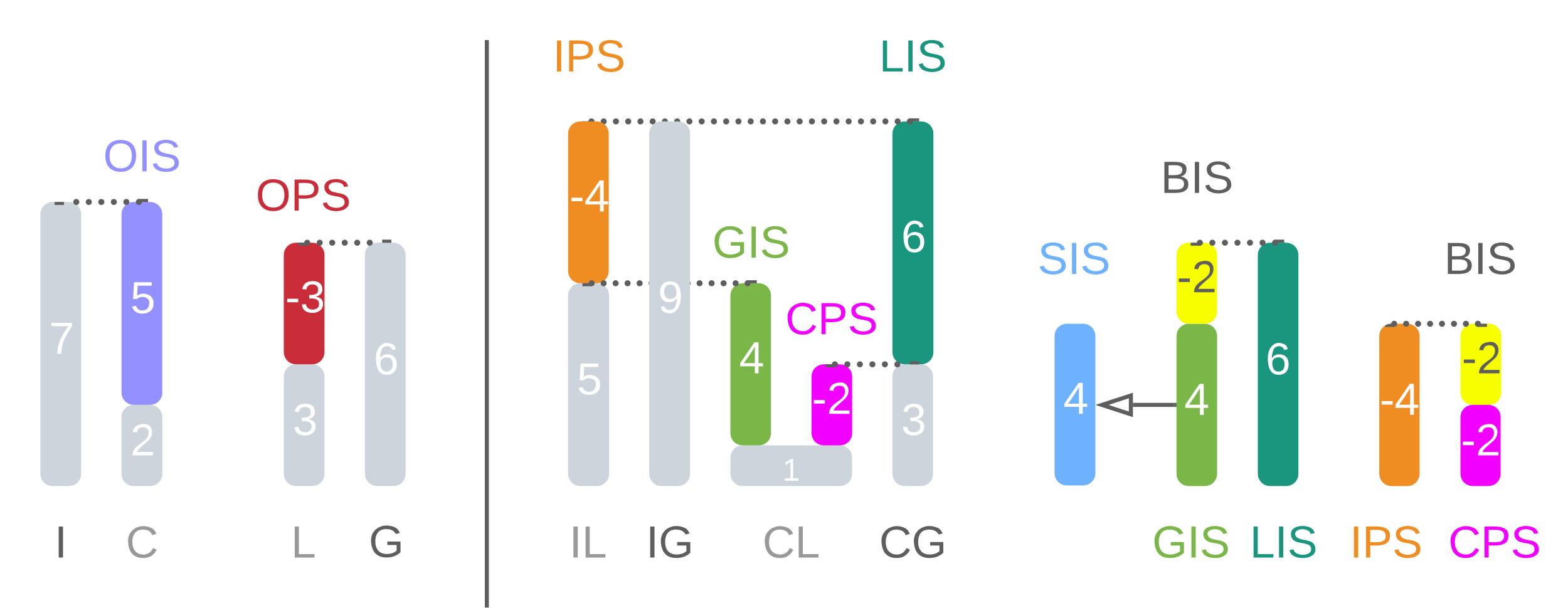


Figure 10: Example calculation of all 8 scores described in Figure 2.

## #3 Effect breakdown

### Interference

In Figure 11 we visualize the relations of the four interference metrics. The plot demonstrates how the shared interference effect (blue) and the biased interference effect (yellow) are positioned in the two-dimensional space created by the local-to-global and global-to-local interference (green) on x and y-axis. Each participant can be projected onto the biased interference by subtracting LIS from GIS (i.e. projecting the participant along the identity line (representing the shared interference) towards the y-axis) and onto the shared interference by using the score with the lower value. This mapping reflects the theoretical background of the interference effect, namely that that incongruent trials are supposed to be slower than congruent trials.


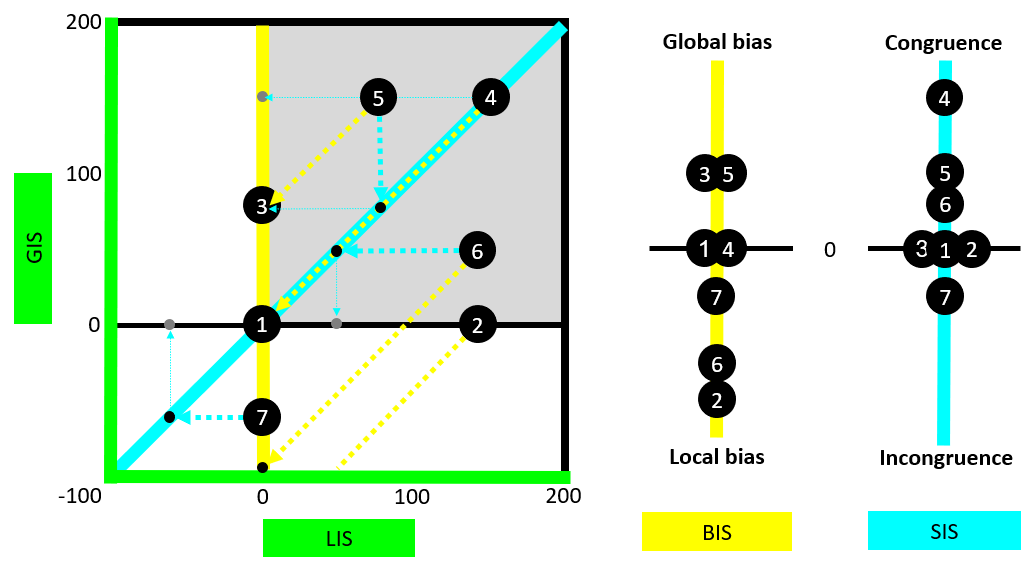


Figure 11: **Interference plot:** Relation of the biased interference (BIS, along the GIS-axis), the shared interference (SIS, identity line), and LIS (x-axis) and GIS (y-axis). Only positive values (marked in grey) are conceptually valid. The calculation of BIS (difference between GIS and LIS) is visualized as yellow arrows pointing *along* the identity line towards the y-axis and thereby makes global-to-local bias positive and local-to-global bias negative (if the arrows would point to the x-axis, the direction of bias would be reversed). The SIS-calculation (effect size shared between GIS and LIS) is visualized as blue arrows pointing to the identity line and then to either the x-axis or y-axis. For a brief interpretation of the seven depicted participants:

1. Neither global-to-local (GIS = 0) nor local-to-global interference (LIS = 0), which means there is neither shared interference nor biased interference (SIS = BIS = 0).
2. Strong local-to-global interference (LIS = 150), but no global-to-local interference (GIS = 0) and thus no shared interference (SIS = 0) i.e., the entire interference is biased (BIS = 150).
3. The exact reverse situation of (2).
4. Equal global-to-local and local-to-global interference (GIS = LIS = 150), which means there is no biased interference, but rather just a strong shared interference effect (SIS = 150).
5. Strong global-to-local interference (GIS = 150) and weak local-to-global interference (LIS = 75) means the interference is split half-half in shared interference (SIS = 75) and global biased interference (BIS = 75).
6. The exact reversed situation of (5)
7. No local-to-global interference (LIS = 0) and a negative global-to-local interference, which means the participant is equally fast in congruent and incongruent global trials and *faster* in incongruent than congruent local trials (global distractor, local target). As negative interference effects are not conceptually sensible, the interpretation and thus calculation is difficult. We apply the same calculation as with positive scores, which means an inversed (negative) global-to-local interference is translated into a local interference bias.

### Precedence

The relation of the three precedence metrics are visualized Figure 12. The principle is the same as in Figure 11, but due to conceptual differences between the factor Congruence and the factor Level the congruent precedence (CPS) doubles as x axis and identity line. Each participant can be projected onto the biased interference by subtracting CPS from IPS (i.e. projecting the participant along the identity line towards the y-axis). Importantly, negative scores are valid in the precedence plot, since they merely denote a local bias, as opposed to a positive global bias.


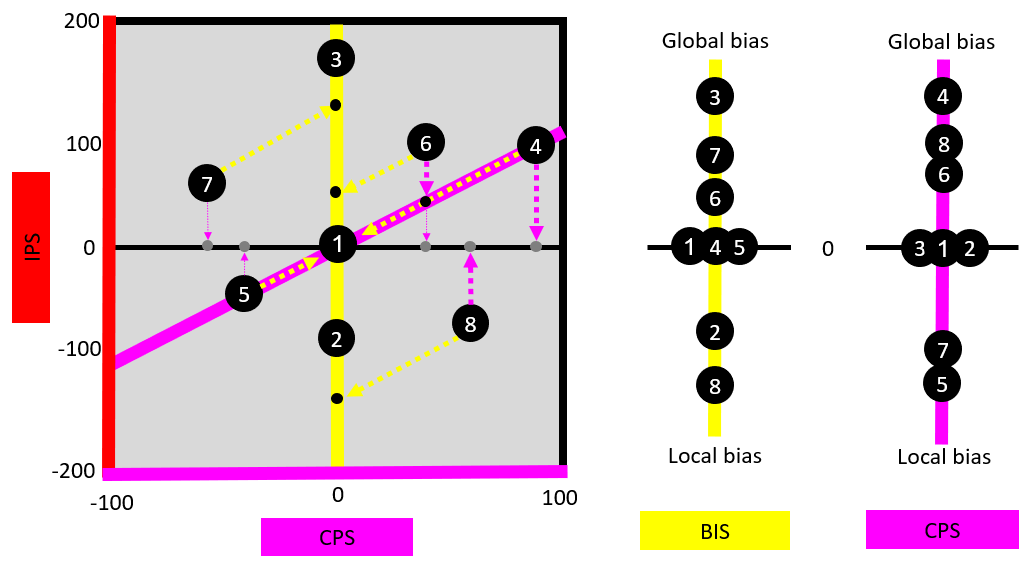


Figure 12: **Precedence plot:** Relation of the biased interference (BIS, along the IPS-axis), the biased precedence (CPS, x-axis and identity line), and IPS (y-axis). All values (positive and negative) are conceptually valid (marked in grey). The calculation of BIS (IPS – CPS) is visualized as yellow arrows pointing along the identity line towards the y-axis. Note that the hypothetical participants in this figure do not correspond to the hypothetical participants in Figure 11.

1. No bias at all, neither in CPS nor in IPS (CPS = IPS = 0).
2. No local-global bias when the figure is congruent (CPS = 0) and thus the bias in incongruent trials is fully caused by the biased interference effect (BIS = IPS = 180).
3. Same as 2 but with negative BIS.
4. The same bias in congruent and incongruent figures (CPS = IPS) which means there is only biased precedence (CPS = 100) and no biased interference effect (BIS = 0).
5. Same as 4, but with negative CPS.
6. Stronger global bias when the figures are incongruent than when they are congruent (IPS=100 > CPS=30). The additional global bias caused by incongruent figures is the biased interference effect (BIS = 70).
7. Local bias in congruent trials (CPS = **-**60), but a weak global bias in incongruent trials (IPS = 20), caused by a local biased precedence effect (CPS = -60) that is overpowered by a strong global biased interference effect (IPS – CPS = BIS=130) in incongruent trials.
8. The exact reversed situation as (7).

## #4 Ratio and scaling

In this article we show the calculation of scores as a simple difference between two conditions (or two other scores in the case of biased interference). We make this choice not only for the sake of simplicity, but also because the two alternatives that have occasionally been used, namely ratio scores or difference-scores that are divided by the participant’s overall reaction time (reaction-time-scaling), seem unnecessary or even counterproductive.

The argument in favour of using ratio-scores or reaction-time-scaling the difference-scores is that with larger reaction times will come larger effect sizes that are outsized compared to the effect sizes of faster participants and do not represent the “true” Navon-effect. This issue could be relevant for the Navon-task, when the difference between two conditions grows with larger overall reaction time. However, the magnitude of this issue is not predetermined and has to be checked in the empirical data itself. It may very well be that this issue only affects a few outliers, as it is not necessarily the case that the effect scales with the overall reaction time. The overall reaction time may be prolonged due to circumstances that are independent from the conditions, in the case of the Navon task for example slower mechanical responses. In that case, scaling the differences-scores with the overall reaction time or using the ratio instead of the difference might falsely reduce the bias strength of slower participants. **Without clear evidence that the strength of biases correlates with the reaction time, non-scaled difference-scores should be used**, as multiple steps of data manipulation can make it harder to correctly assess and interpret the results.

Beyond the above argument, ratio-scores should never be used in Navon, because both conditions (e.g. local trials vs. global trials) are of the same “type”, but in a ratio the denominator and enumerator have different mathematical meaning that is inseparable from the resulting ratio: dividing 4 by 5 or 5 by 4 does not result in the same *absolute* ratio (0.2 and 0.25, respectively). Thus, the **ratio distorts the bias scores of some participants in relation to the scores of the other participants**, unless all scores of one type were larger than the scores of the other type.

For these reasons we argue that simple difference-scores should be used. Scaling by dividing (or preferable regressing) the score to the (overall) reaction time should only be done if the data clearly shows that it is necessary to prevent a significant distortion of the results.

## #5 Alternative measures

The scores described in Figure 2 can be calculated with any measure: reaction time, error rates, combined measures (like the inverse efficiency score), or even neurometric measures like EEG data. However, the conceptual and theoretical meaning of the resulting metrics may differ greatly from the classical interpretation of the mean reaction time.

For example, in CPS, slower processing of information (mean reaction time) from one level than the other level is clearly interpretable as a bias, while more errors (mean accuracy) in one level than the other is not as clearly interpretable as bias, because in the current framework, errors in congruent trials should be random errors, not systematic errors, as only the correct answer is visible on screen. The theoretical framework supporting an accuracy-based CPS would thus need to reflect that bias could somehow introduce more premature (and thus random) answers. An accuracy-based IPS on the other hand is comparable to the reasoning behind reaction-time biases.

This example demonstrates the need to consider the conceptual and theoretical implication of measure-metric interactions carefully.

## #6 Meta analyses


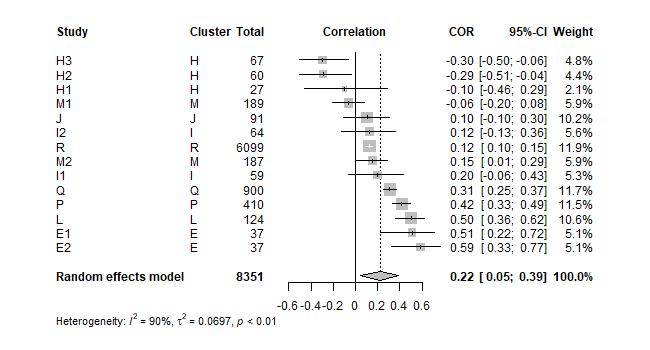


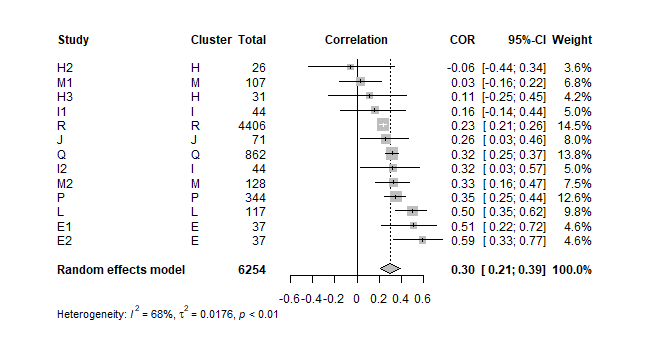


Figure 13: Split-half reliability of all GIS (top) and only non-negative GIS (bottom) in those groupings that were provided as trial-data.


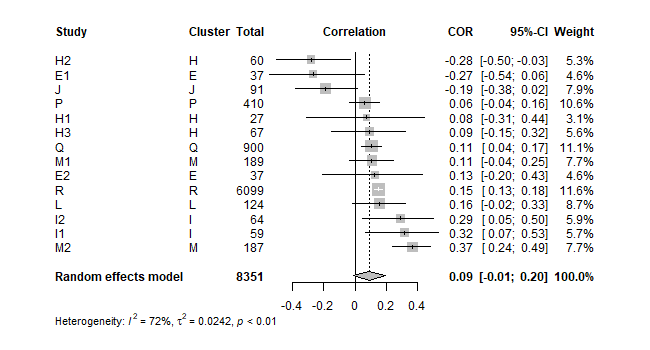

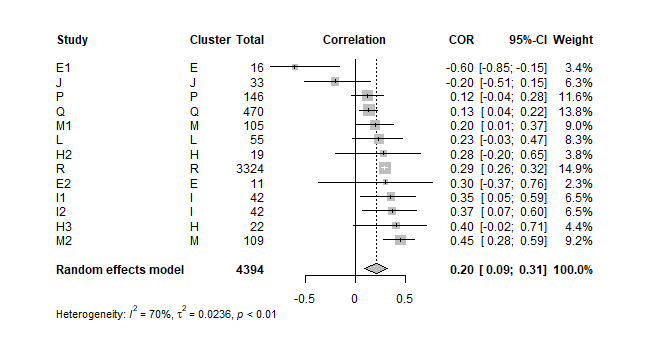


Figure 14: Split-half reliability of all LIS (top) and only non-negative LIS (bottom) in those groupings that were provided as trial-data.


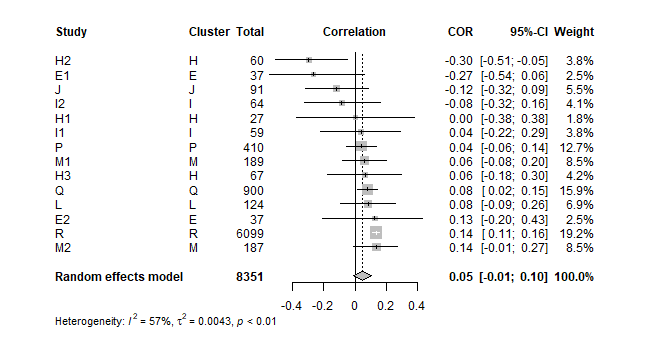

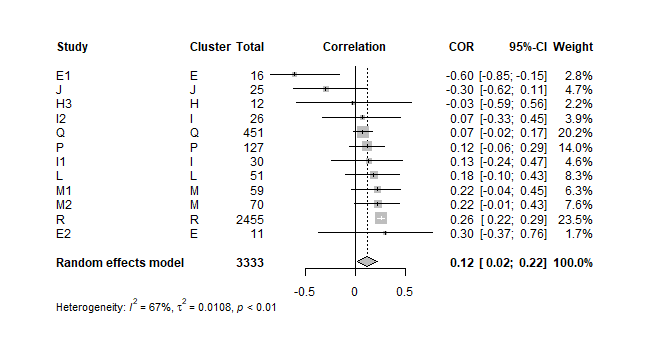


Figure 15: Split-half reliability of all SIS (top) and only non-negative SIS (bottom) in those groupings that were provided as trial-data.


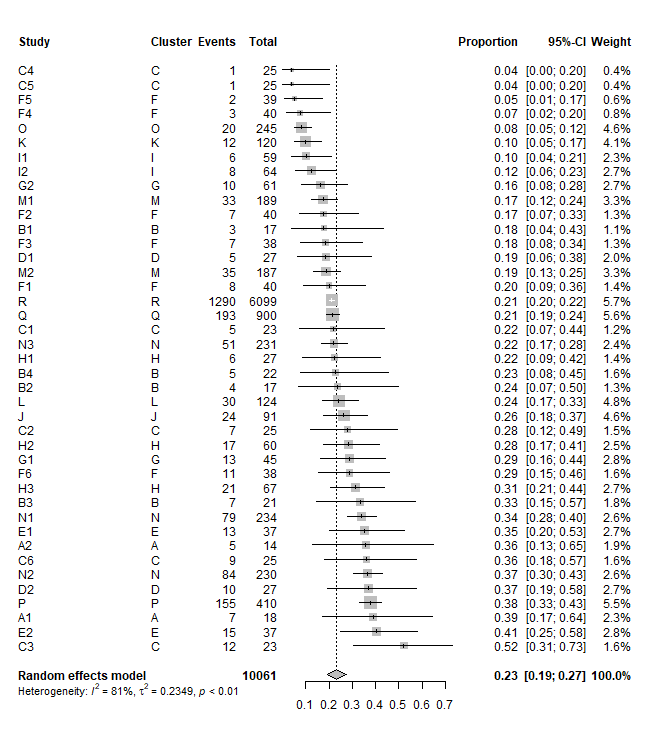


Figure 16: Forest plot of the proportion of negative LIS in all groupings.


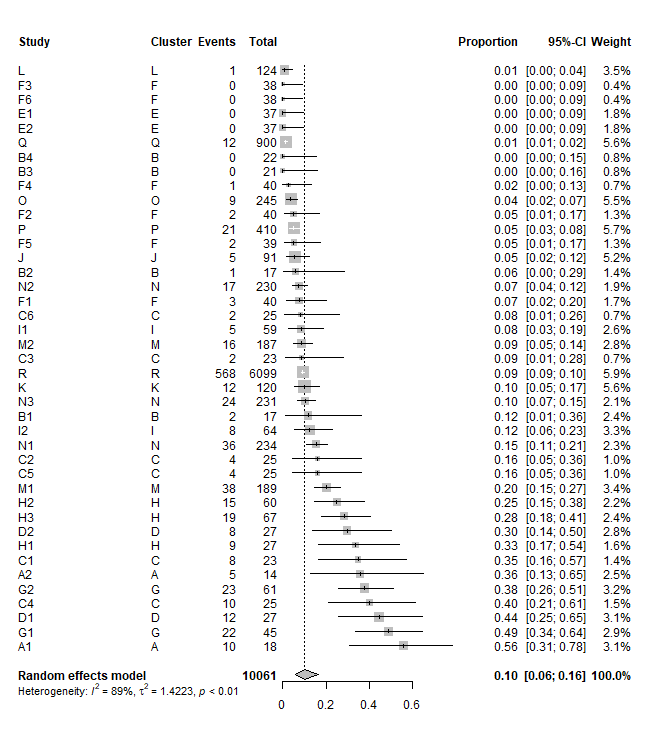


Figure 17: Forest plot of the proportion of negative GIS in all groupings.


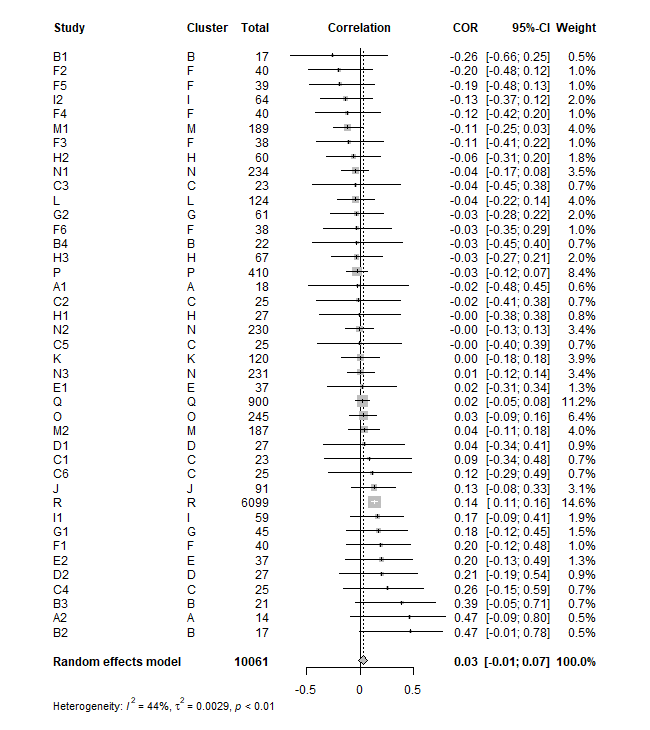


Figure 18: Forest plot of the correlation between GIS and LIS in all groupings.


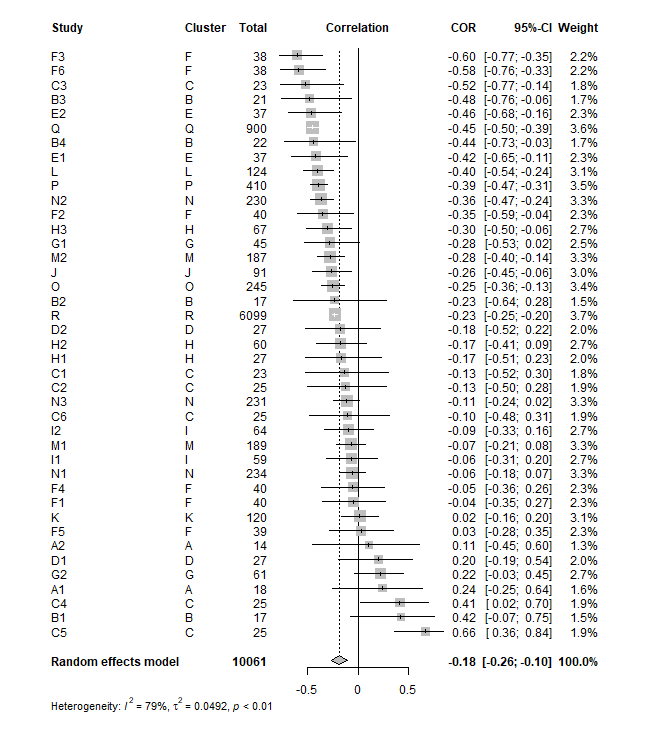


Figure 19: Forest plot of the correlation between BIS and SIS in all groupings.


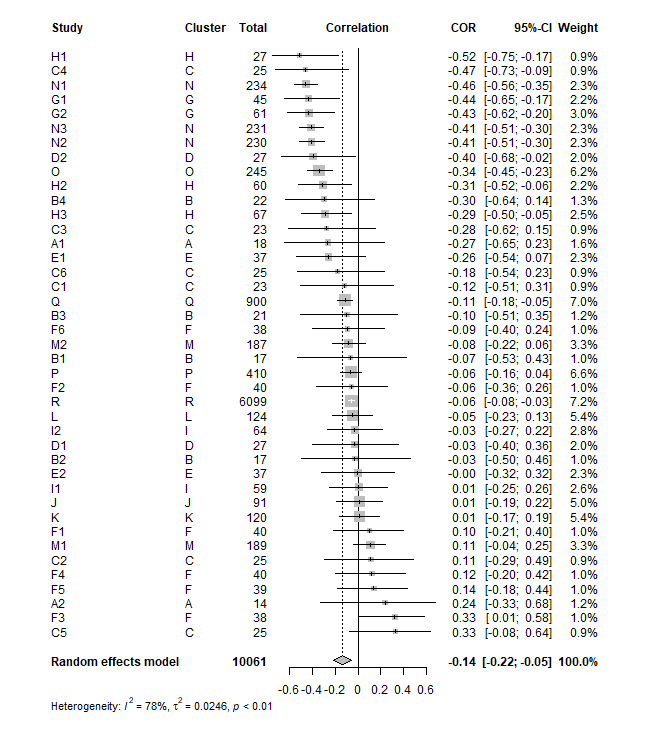


Figure 20: Forest plot of the correlation between BIS and CPS in all groupings.

## #7 Split-half reliability


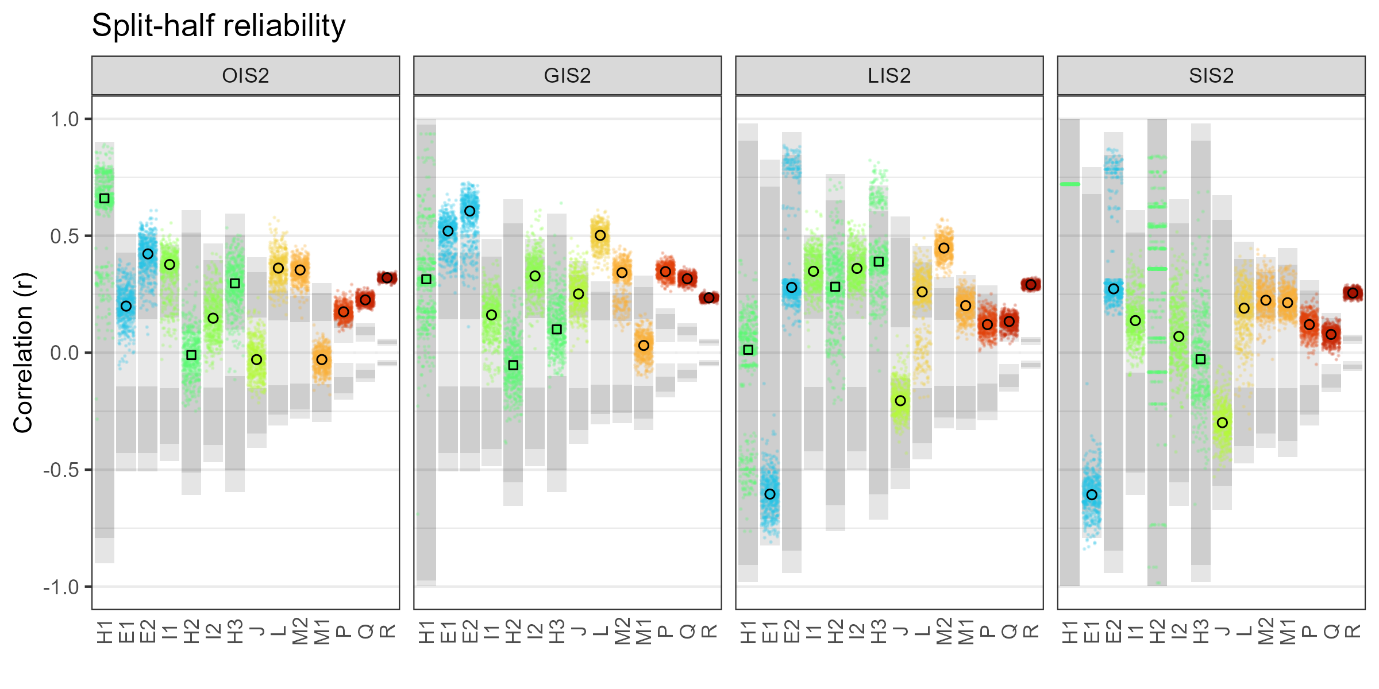


Figure 21: Split-half reliability of non-negative interference scores. Due to lower participant numbers the range of indecisive bayes factors (grey) is wider than for correlations of all participants.


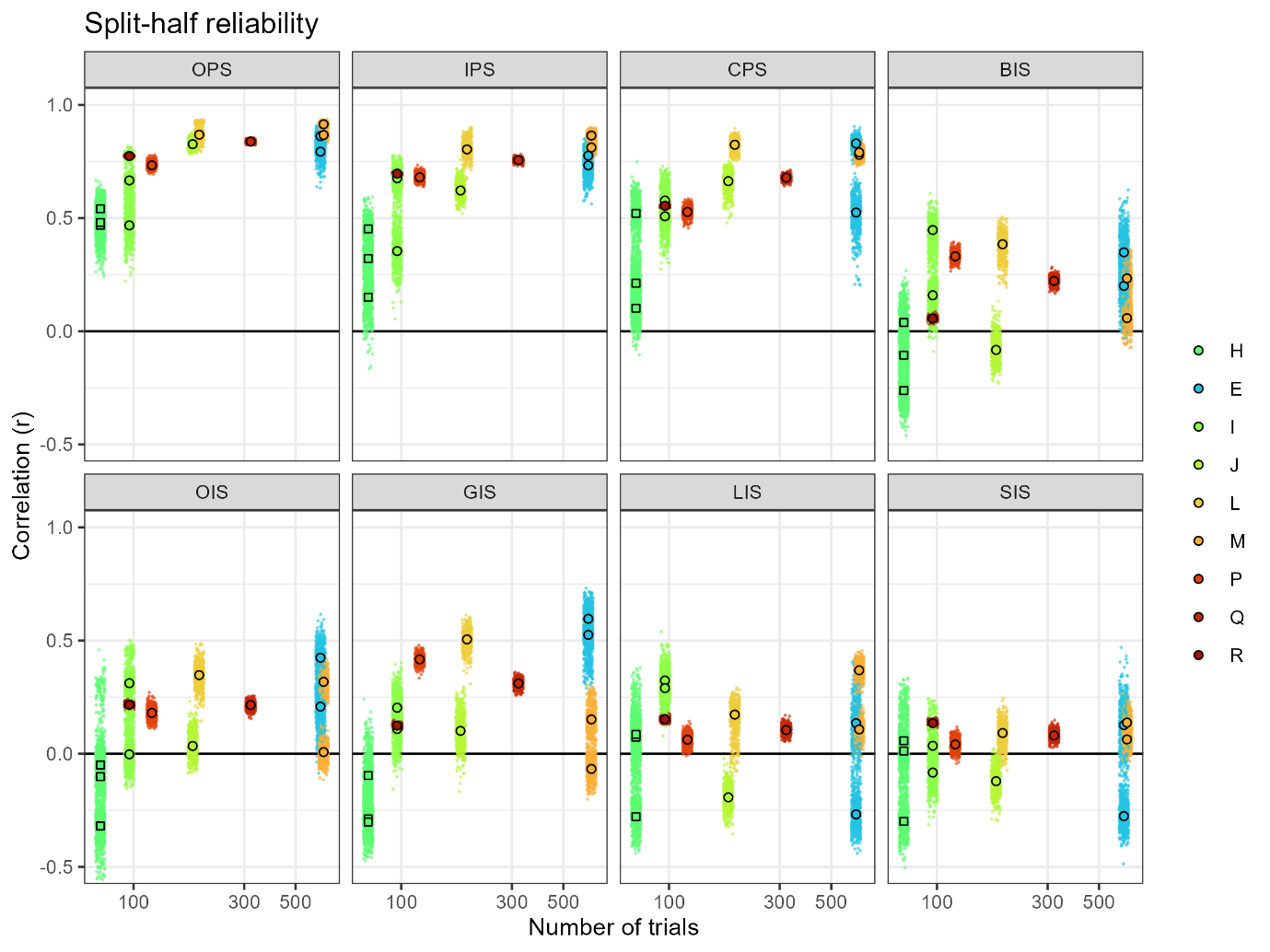


Figure 22: Split-half reliability of all groupings plotted against the number of trials. The small dots represent 500 bootstrapped Pearson correlations, each with 80% of the grouping’s participants, the larger dots their median. The colour-ordering indicates the participant number.

### Reliability correction

When interpreting the correlation between two metrics, the metrics’ internal reliability should be considered. A metrics with low reliability is more likely to produce weak correlations. One can postulate that the metric captures a true and singular effect that is masked by small noise-effects (either measurement errors (regression dilution) or conceptually relevant effects) and that the *effect’s* (latent variable) “true correlation” is higher than the *metric’s* (observed variable) “noisy correlation”. One method to correct for this is to divide the correlation by the square root of the product of the split-half reliability of the two correlated metrics.

$$\frac{r_{correlation}}{\sqrt{r_{reliabilIty 1}*r_{reliability 2}}}$$

However, when the reliabilities are low, this method can lead to very strong corrections far beyond r = 1 and correlations become much more difficult to interpret. Furthermore, negative split-half reliabilities cannot be used, as they would flip the direction of the correlation. Lastly, if the “noise” in the metric is not due to measurement errors but rather the absence or weakness of a clear primary effect, this correction artificially inflates correlation strengths. We therefore show non-corrected correlations throughout the paper. For the datasets with trial-data, we show the reliability-corrected correlations in Figure 24. The resulting values vary wildly and are difficult to interpret.

## #8 More correlations


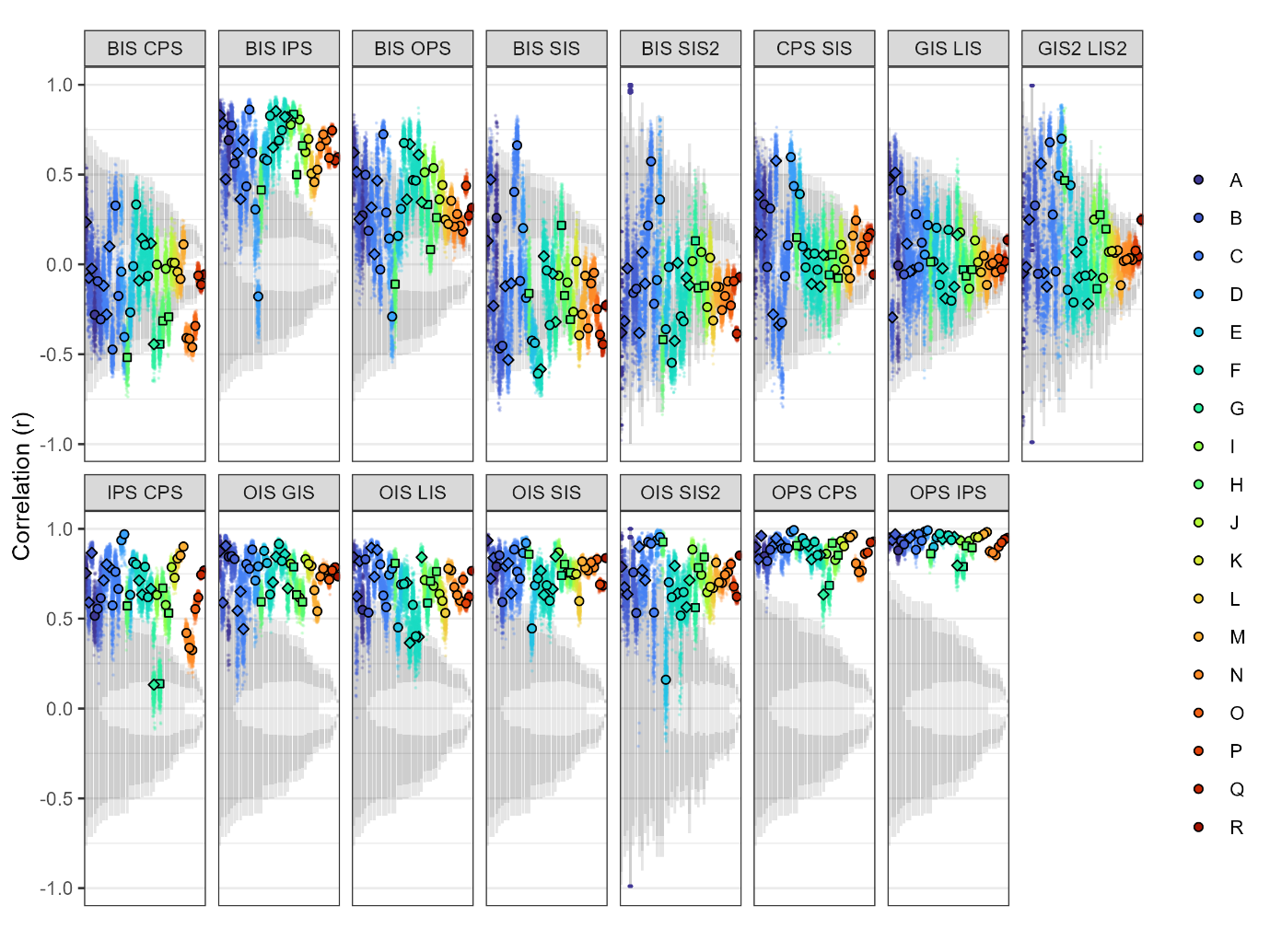


Figure 23: Conceptually relevant Pearson correlations between metrics (meta analyses in <https://osf.io/gtqu8/>). The small dots represent 500 bootstrapped Pearson correlations, each with 80% of the grouping’s participants, the larger dots their median. The shaded area indicates the negative and positive range of Pearson r which – for a given participant number – correspond to Bayes factors between 0.1 and 0.3 (inner light shade, moderate evidence for non-correlation), 0.3 and 3 (dark shade, uncertain evidence), and 3 and 10 (outer light shade, moderate evidence for correlation). In GIS2, LIS2, and SIS2, all participants with negative values were removed. Note that not all metrics are independent, for example the data of CPS makes up half the data for OPS (with IPS making up the other half). However, these correlations are practically relevant, to assess how the results of a study using OPS would translate to using CPS instead. Fully data-independent correlations are thus only IPS-CPS and GIS-LIS. However, conceptually sensible correlations can also be based on the same data, when the data is processed in such a way that separate effects are extracted.


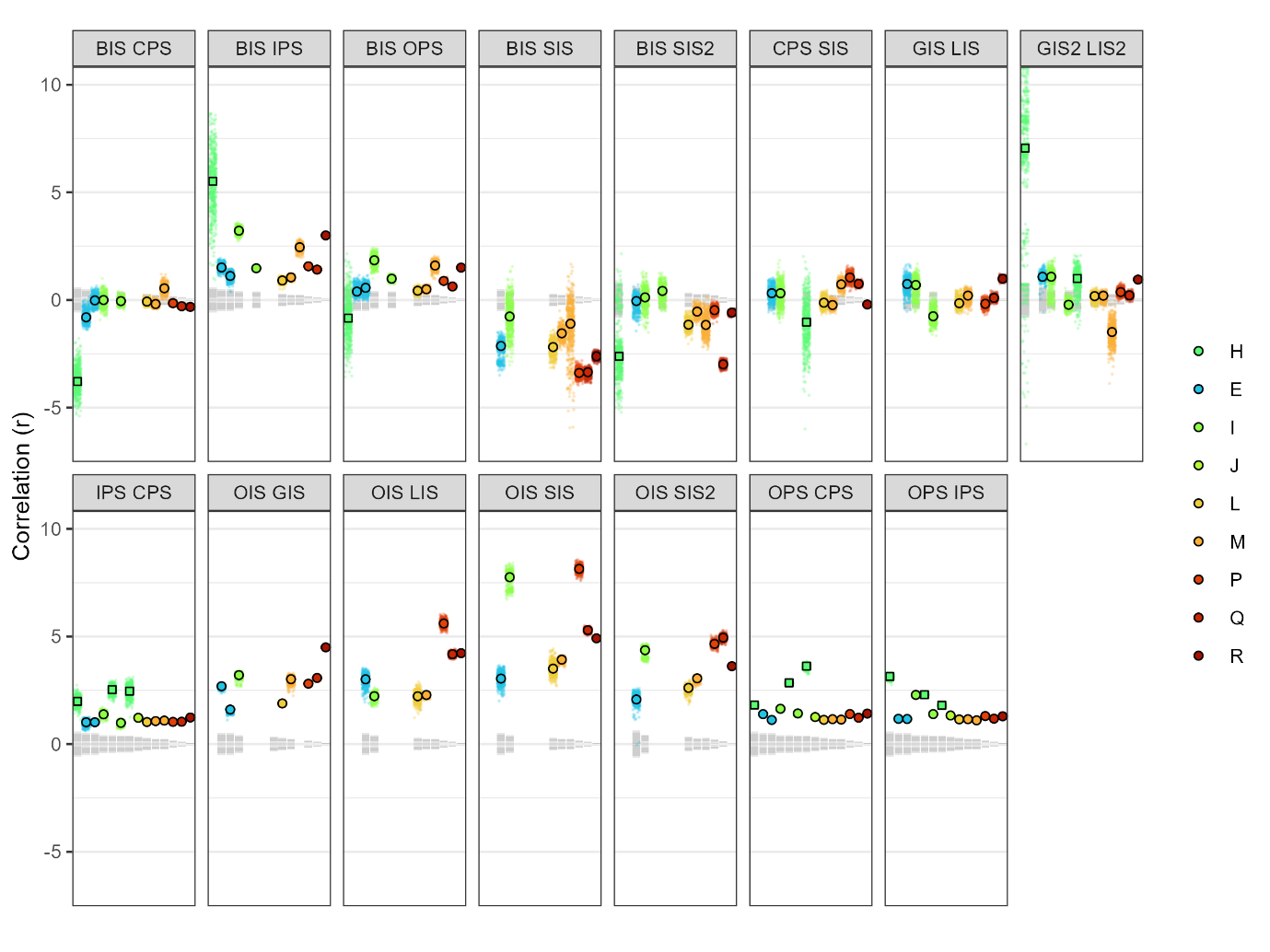


Figure 24: Correlations between metrics as in Figure 23, but corrected for split-half reliabilities using the formula mentioned in Appendix #7. Missing correlations are due to negative split-half reliabilities that cannot be used for corrections.


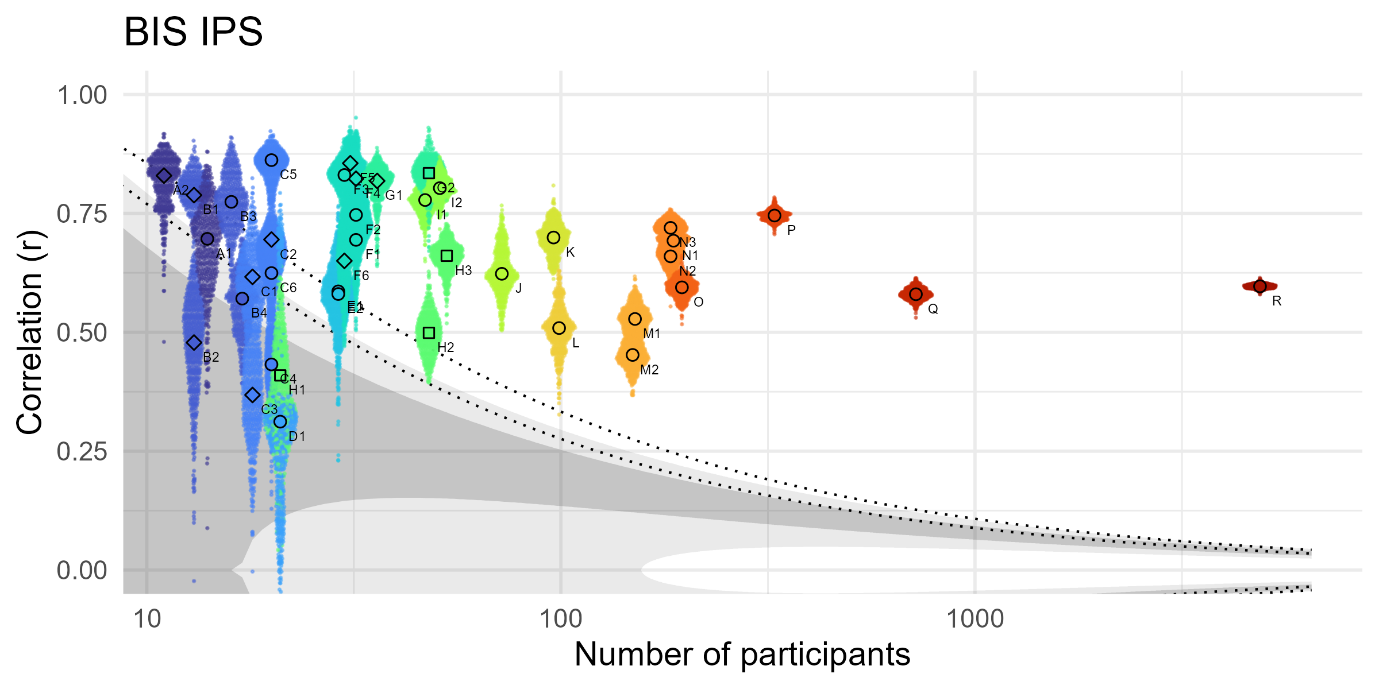


Figure 25: Correlation between BIS and IPS. The concept of the figure is the same as Figure 7, Figure 8, and Figure 9. Grouping D2 has a median negative correlation of about 0.2 and is removed for visualization purposes.


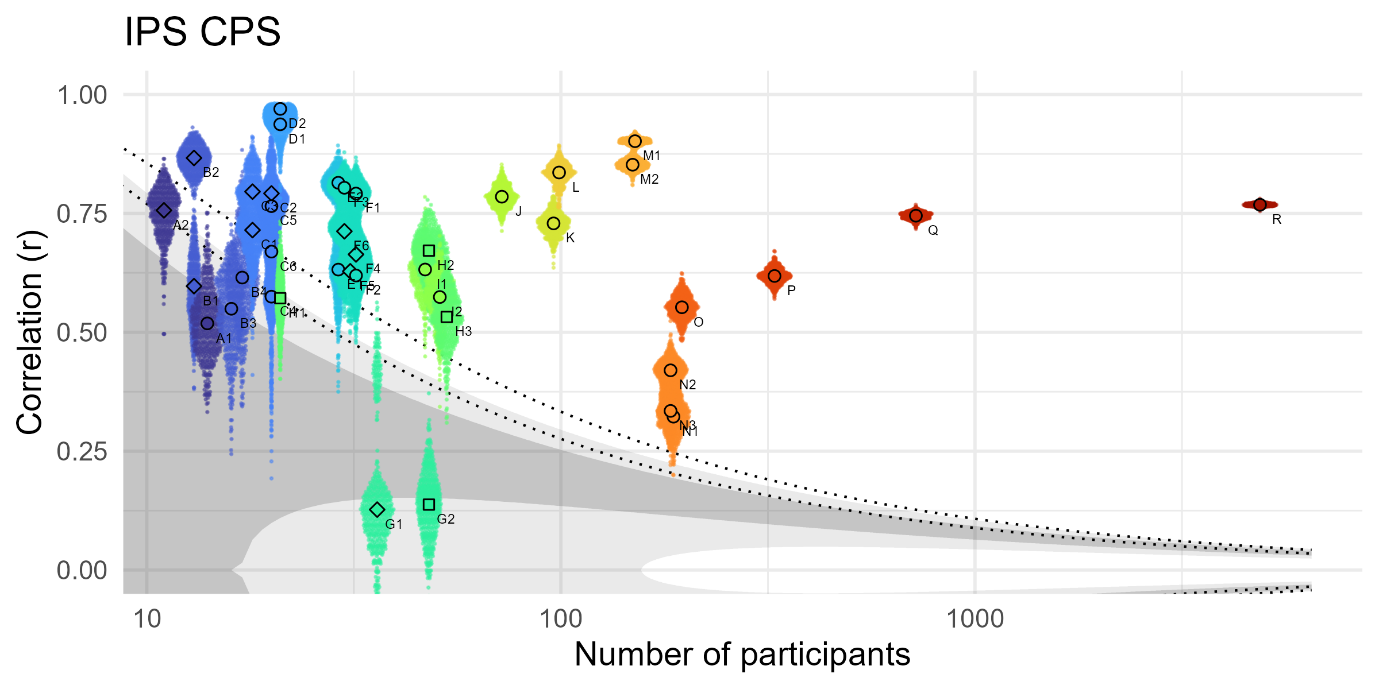


Figure 26: Correlation between IPS and CPS. The concept of the figure is the same as Figure 7, Figure 8, and Figure 9.


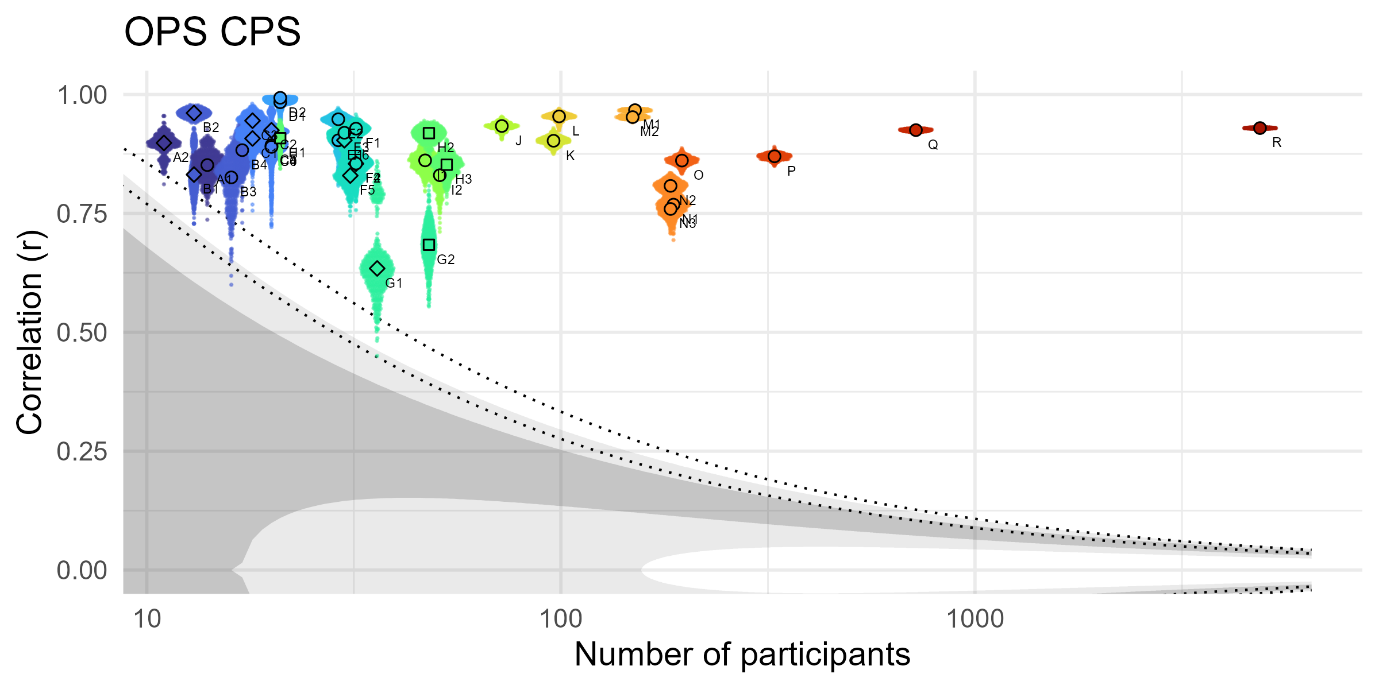


Figure 27: Correlation between OPS and CPS. The concept of the figure is the same as Figure 7, Figure 8, and Figure 9.


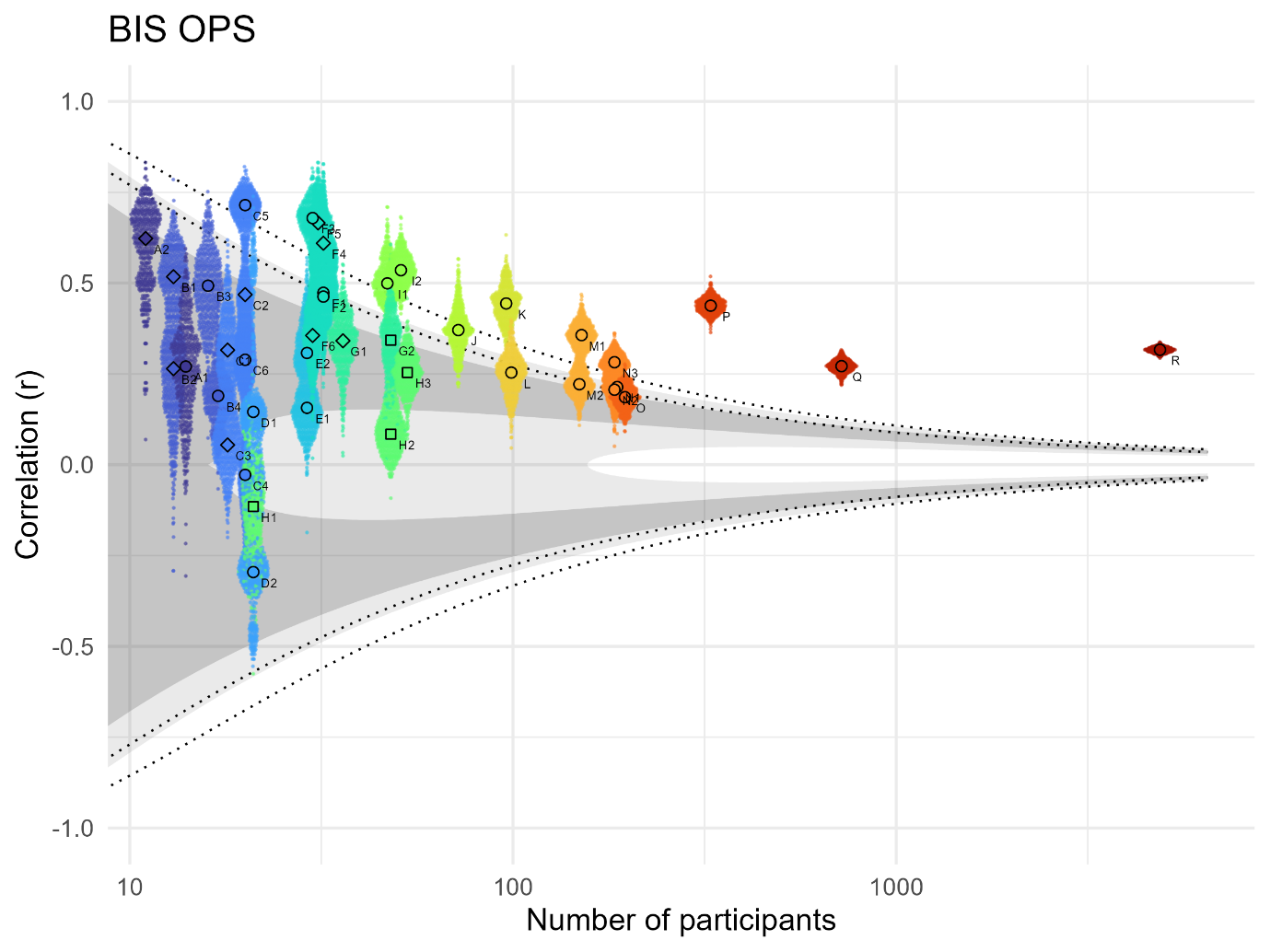


Figure 28: Correlation between BIS and OPS. The concept of the figure is the same as Figure 7, Figure 8, and Figure 9.


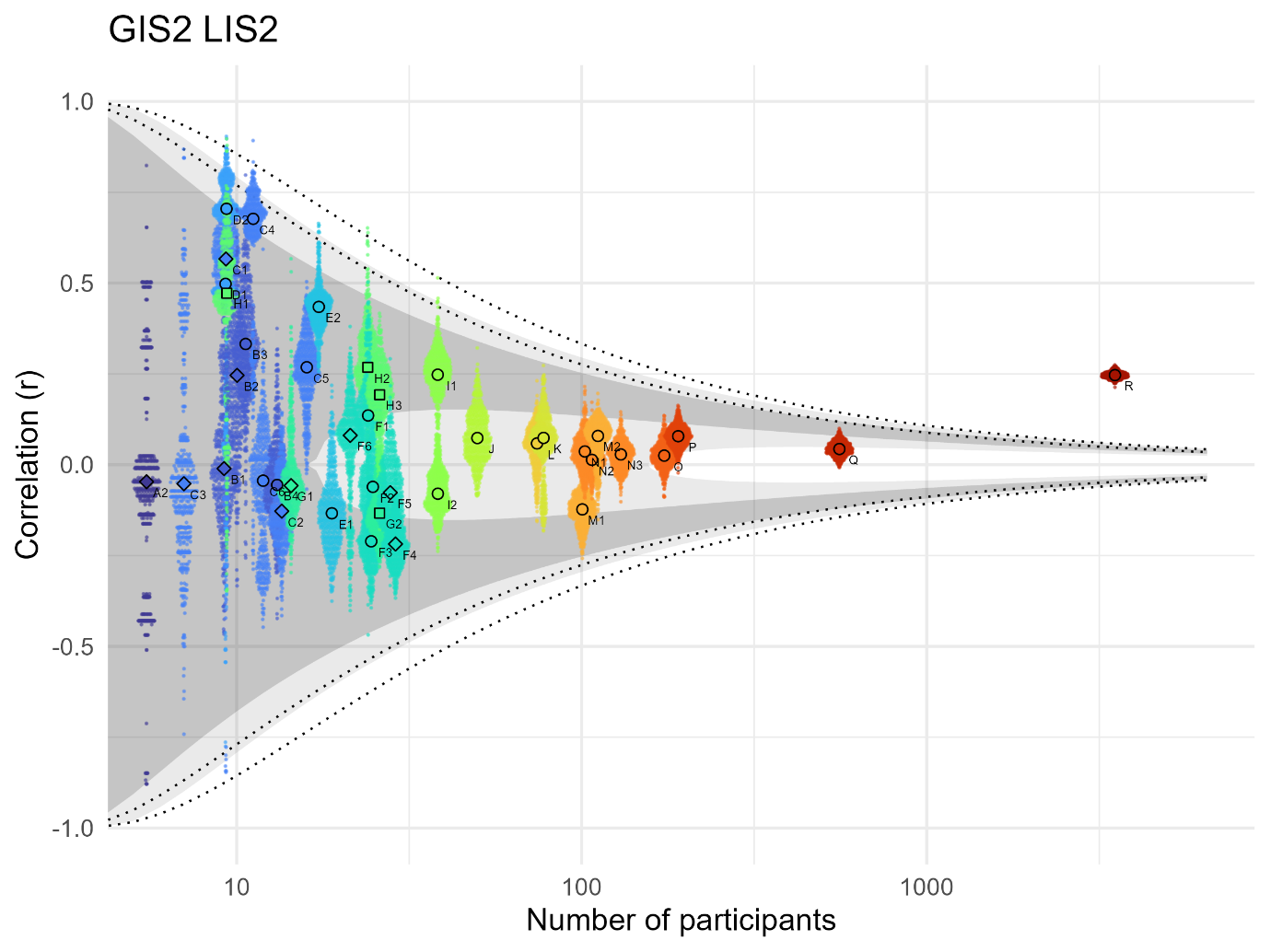


Figure 29: Correlation between GIS2 and LIS2, i.e. GIS and LIS without participants with negative scores. Participant numbers are thus significantly smaller. The concept of the figure is the same as Figure 7, Figure 8, and Figure 9.

## #9 LIS-GIS distributions

We can attempt to infer the existence of opposite effects from the spread of the GIS and LIS distribution by using the following three premises:

1. The spread of a distribution is determined firstly by the individual difference in effect strength (and direction) and secondly by effect-independent noise.
2. A distribution that is shifted from 0 is certain to contain an effect in addition to noise, as noise would be distributed around 0.
3. LIS distributions that are close to 0 still have a similar spread to their respective GIS distributions that are shifted much further from 0 (Figure 30).

Based on these statistical and empirical premises, one could argue that since the spread of GIS is only partially caused by noise and possibly even mainly by the effect(s) captured by GIS, the same should apply to LIS, i.e. that true effects should exist in LIS, not only noise. In order for LIS to be close to 0, there would need to be at least to latent effects that cancel each other out. In the case of LIS we can conceptually infer that these effects would be congruent interference on one hand and incongruent interference, congruent facilitation, or both on the other hand (see Figure 3). However, this entire line of reasoning is highly speculative.


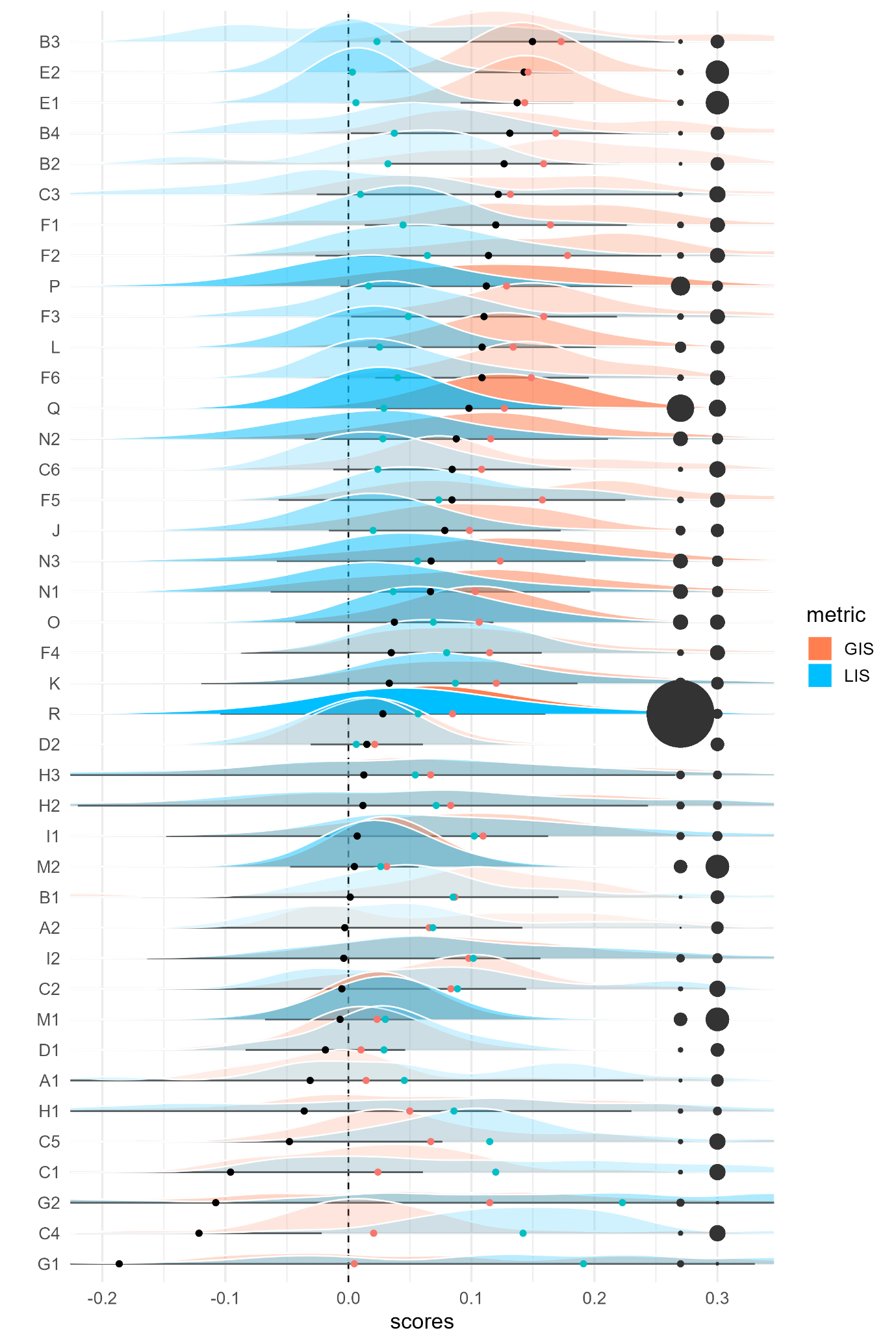


Figure 30: GIS and LIS density probability function and median scores in colour. In black, the mean and standard deviation of participants’ difference between GIS and LIS (i.e., the BIS). For visualization purposes, participants’ GIS, LIS, and BIS were scaled to the mean reaction time of their dataset. The two columns of dots on the right represent participant number (left column) and trial number (right column), respectively. No clear relation of these two values and the GIS-LIS distribution is visible. While there is a general trend towards a stronger GIS and weaker LIS, this differs between datasets and some datasets. In dataset C, large local elements (C1, C4) cause stronger LIS and weaker GIS than medium local elements (C2, C5) or even small elements (C3, C6) (Baisa et al., 2021). Datasets L, J, and D show quite strongly overlapping GIS and LIS with a comparatively narrow spread, while other datasets, especially E, show clear GIS but weak LIS. These patterns are also reflected in the split-half reliability, where dataset datasets Q, N, and K have a comparatively strong split-half reliability GIS and also comparatively strong GIS distribution. Dataset L has a comparatively stronger split-half reliability for LIS than GIS and a comparatively weak GIS distribution. While there seem to be patterns in these distributions, the current analysis is not sufficient to draw clear conclusions.


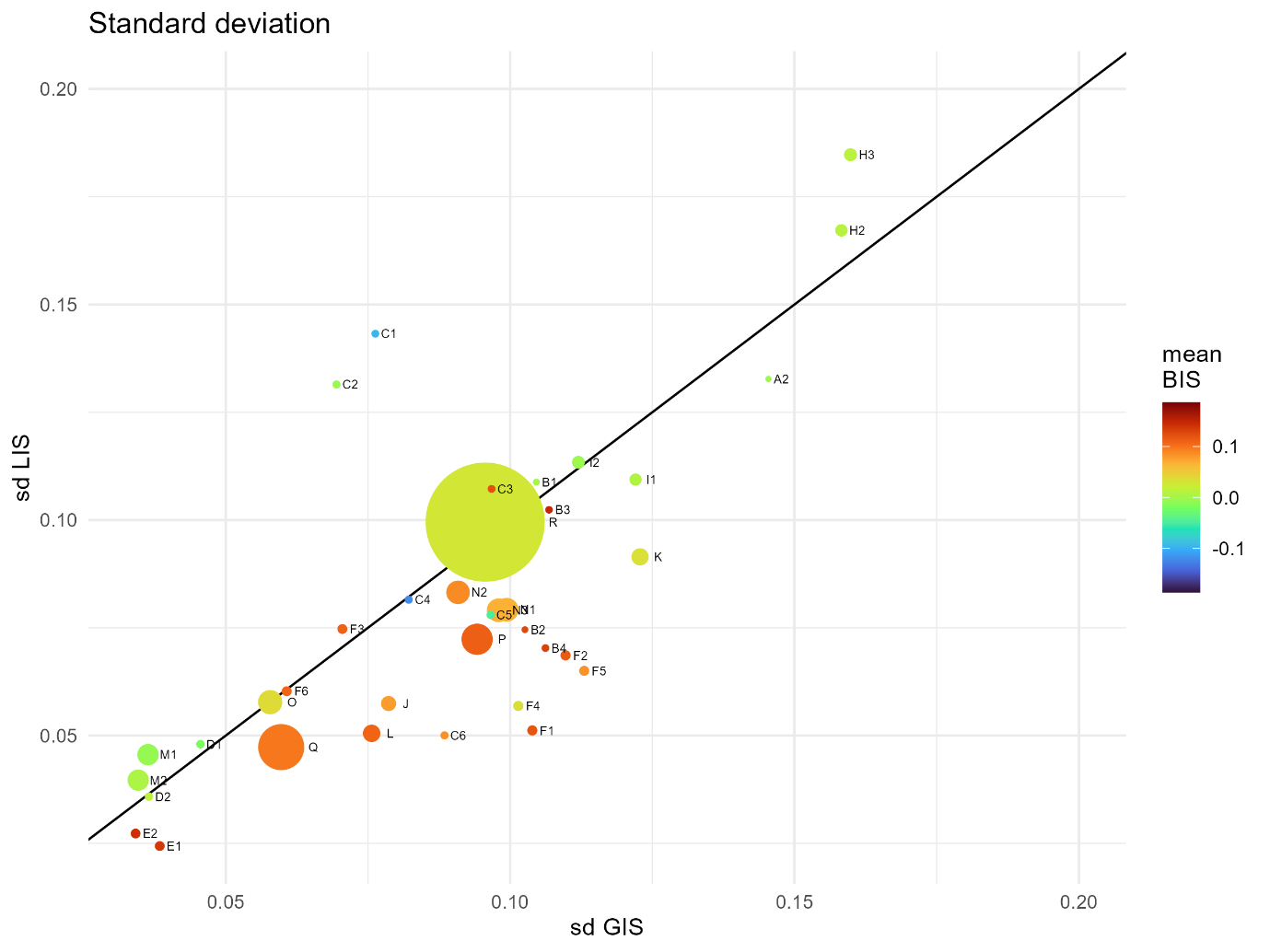


Figure 31: Spread of GIS and LIS in each grouping plotted against each other. The colour indicates whether the grouping has a positive or negative population bias for BIS. In black, the identity line showing a hypothetical perfect correlation, with the empirical data being fairly close. Three groupings with a much wider spread (but still close to the identity line) in both metrics are omitted for visualization purposes (A1, G1, G2, H1).
